# Cooperative stabilisation of 14-3-3σ protein–protein interactions *via* covalent protein modification†

**DOI:** 10.1039/d1sc02120f

**Published:** 2021-09-06

**Authors:** Marta Falcicchio, Jake A. Ward, Sara Y. Chothia, Jaswir Basran, Alisha Mohindra, Salvador Macip, Pietro Roversi, Richard G. Doveston

**Affiliations:** Leicester Institute for Structural and Chemical Biology, University of Leicester University Road Leicester LE1 7RH UK r.g.doveston@leicester.ac.uk; School of Chemistry, University of Leicester University Road Leicester LE1 7RH UK; Mechanisms of Cancer and Aging Laboratory, Department of Molecular and Cell Biology, University of Leicester University Road Leicester LE1 7RH UK; Department of Molecular and Cell Biology, University of Leicester University Road Leicester LE1 7RH UK; FoodLab, Faculty of Health Sciences, Universitat Oberta de Catalunya Barcelona Spain; Institute of Agricultural Biology and Biotechnology, IBBA-CNR Unit of Milano Via Bassini 15 I-20133 Milan Italy

## Abstract

14-3-3 proteins are an important family of hub proteins that play important roles in many cellular processes *via* a large network of interactions with partner proteins. Many of these protein–protein interactions (PPI) are implicated in human diseases such as cancer and neurodegeneration. The stabilisation of selected 14-3-3 PPIs using drug-like ‘molecular glues’ is a novel therapeutic strategy with high potential. However, the examples reported to date have a number of drawbacks in terms of selectivity and potency. Here, we report that WR-1065, the active species of the approved drug amifostine, covalently modifies 14-3-3σ at an isoform-unique cysteine residue, Cys38. This modification leads to isoform-specific stabilisation of two 14-3-3σ PPIs in a manner that is cooperative with a well characterised molecular glue, fusicoccin A. Our findings reveal a novel stabilisation mechanism for 14-3-3σ, an isoform with particular involvement in cancer pathways. This mechanism can be exploited to harness the enhanced potency conveyed by covalent drug molecules and dual ligand cooperativity. This is demonstrated in two cancer cell lines whereby the cooperative behaviour of fusicoccin A and WR-1065 leads to enhanced efficacy for inducing cell death and attenuating cell growth.

## Abstract 2

The aminothiol WR-1065 covalently modifies 14-3-3σ to stabilse its interactions with p53 and ERα. It enhances the effect of fusicoccin A *via* a cooperative mechanism that leads to 14-3-3 partner-protein specific activty against cancer cells.

## Graphical abstract

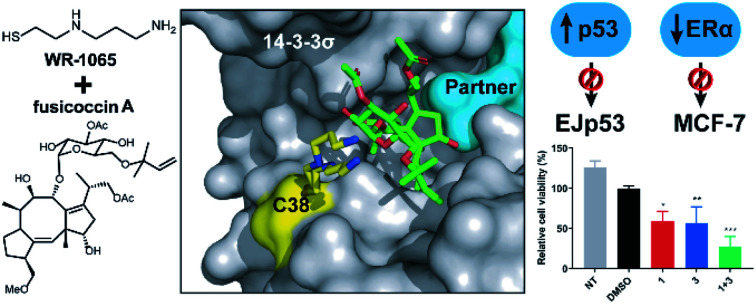

## Introduction

The 14-3-3 family of hub proteins (seven isoforms: β, γ, ε, η, σ, τ, ζ) play diverse and important roles in maintaining normal cell function through interaction with over 200 partner proteins.^1,2^ These protein–protein interactions (PPIs) are typically dependent on the phosphorylation of specific recognition motifs within disordered domains of the partner protein. Through these PPIs, 14-3-3 proteins modulate the subcellular localisation, protein folding, enzymatic activity or interaction profile of their partners.^3^ Many 14-3-3 PPIs are implicated in human diseases, for example those in which there is an involvement of Raf kinases (cancer),^4^ Cdc25 (cancer),^5^ CFTR (cystic fibrosis),^6^ tau (Alzheimer's disease),^7^ LRRK2 (Parkinson's disease),^8^ ERα (breast cancer)^9^ and p53 (cancer).^10^ Thus, 14-3-3 PPIs have attracted increasing interest as potential drug targets.^1^ In particular, the stabilisation of certain 14-3-3 PPIs has enormous potential because of the desirable therapeutic benefits in anti-cancer therapy. Stabilisation of PPIs also presents an opportunity to selectively target unique protein–protein interfaces because shape complementarity is required with both protein partners. Selective inhibition in this context poses the much greater challenge.

**Fig. 1:**
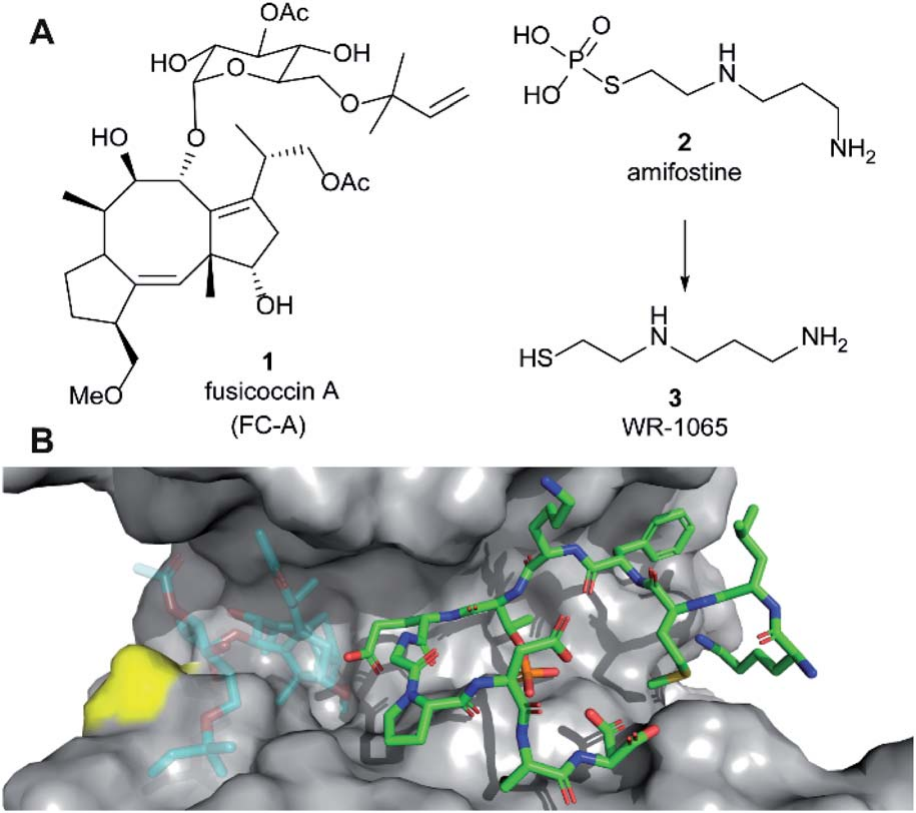
(A) Structures of fusicoccin A (**1**, FC-A), amifostine (**2**) and WR-1065 (**3**). (B) Crystal structure showing a phospho-peptide representing the C-terminus of p53 (pT387, stick representation, C atoms in green) in complex with 14-3-3σ (grey). The position of Cys38 is indicated in yellow. PDB: 5MOC.^10^ The binding site of **1** is indicated by the structure of **1** shown in faded colours (overlaid from PDB: 5MXO).^10^

Examples of small molecule PPI stabilisers remain rare,^11^ and the cooperative nature of ternary complex formation presents a challenge in terms of ligand discovery and optimisation.^12^ A limited number of 14-3-3 PPI stabilisers have been reported, the majority of which target, or are predicted to target, pockets at the protein–protein interface within the amphipathic binding groove of 14-3-3.^1^ Examples include the fusicoccane family of diterpene natural products (*e.g.***1**, fusicoccin A, Fig. 1A),^13^ epibestatin,^14^ pyrrolidone,^14^ pyrazole,^15^ and supramolecular ligands.^16^ These compounds have a number of drawbacks in terms of synthetic tractability, drug-likeness, potency and/or selectivity. Furthermore, they do not distinguish between the seven human 14-3-3 isoforms that each have distinct roles. For example, gathering structure–activity relationship data around the fusicoccane family requires complex semi-synthesis, and the most widely available member, **1**, targets all 14-3-3 isoforms and at least three different binding interfaces (ERα, CFTR and p53). Therefore, it is important to identify and rationally develop novel 14-3-3 stabilisation mechanisms that may circumvent such drawbacks.

Amifostine (**2**) (Fig. 1A) is a clinically approved prodrug administered to alleviate side effects associated with chemotherapy and radiotherapy.^17^*In vivo*, **2** is dephosphorylated to release the active species, aminothiol WR-1065 (**3**, Fig. 1A). **3** exerts its effects through a number of possible mechanisms, most predominant of which is the scavenging of harmful reactive oxygen species.^17^ Notably, **3** is known to modulate the activity of the transcription factors NFkB, AP-1 and p53 *via* covalent disulphide bond formation in cellular environments.^18^ There is also compelling evidence that **3** enhances wild-type p53 activity^19^ and rescues the activity of p53 mutants by modulating protein conformation.^20^ Furthermore, **2** promotes the interaction of p53 with 14-3-3σ presumably *via***3** as the active species.^21^ This PPI preserves p53 protein levels in the cell and enhances its transcriptional activity.^21,22^ p53 interacts with 14-3-3σ *via* one or more phosphorylated motifs in its disordered C-terminal domain (*e.g.* pT387, Fig. 1B).^22^ To date, only the p53 DNA-binding domain has been associated with covalent modification by drug molecules.^23^ Therefore, current evidence does not point to any clear rationale for how **3** could directly influence the p53 C-terminal domain interaction with 14-3-3σ PPI.

Here we report that **3** stabilises two distinct 14-3-3σ PPIs (with p53 and ERα) *via* covalent protein modification of 14-3-3σ at Cys38, a solvent exposed residue unique to this human 14-3-3 isoform (Fig. 1B). It exerts its effect *via* a mechanism that also enhances the effect of the known stabiliser **1** in a cooperative manner. The stabilisation effect is not specific to a single 14-3-3σ PPI, but it is specific to the sigma isoform *in vitro*. Our findings demonstrate a new mechanism for 14-3-3σ stabilisation that can harness the enhanced potency of covalent drugs, and combination treatment, to achieve a desirable therapeutic response. The enhanced efficacy of combination treatment of **1** and **3** against cancer is demonstrated in two cancer cell models. Combination treatment more effectively induced a p53-specific senolytic effect on senescent EJp53 cells and was more effective at attenuating the growth of estrogen-induced MCF-7 cell growth. Our results also highlight a new mechanism that can ultimately be used to achieve 14-3-3σ isoform specificity, and is likely to contribute to the *in vivo* pharmacodynamics of **2**.

## Results and discussion

### WR-1065 covalently modifies 14-3-3σ Cys38

**Fig. 2:**
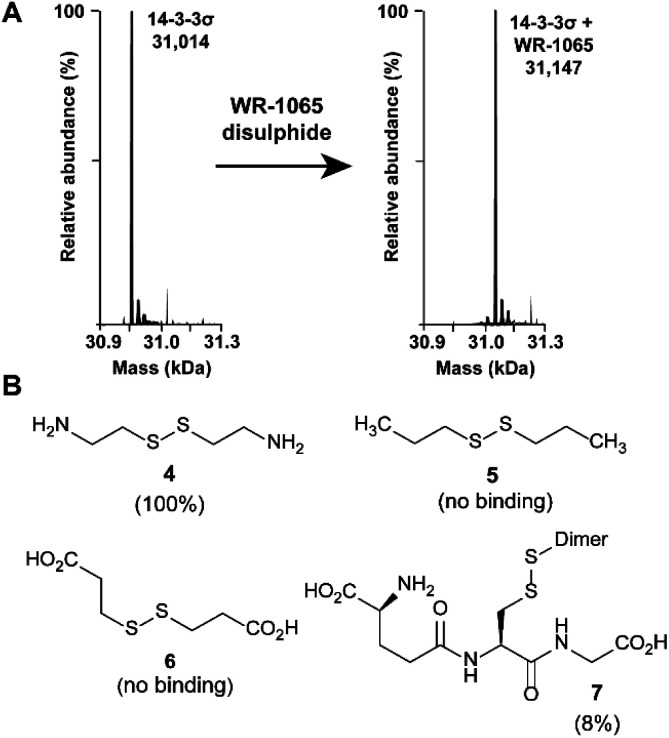
Amino disulphides covalently modify 14-3-3σ. (A) Deconvoluted mass spectra for 14-3-3σ before and after modification by the homodisulphide of **3**. (B) Structures of disulphide-containing molecules screened for binding to 14-3-3σ (% ligation is given in parentheses).

Because **3** had previously been shown to elicit a biological response through covalent modification of target proteins, we first used mass spectrometry to establish if it also reacted with Cys38 of 14-3-3σ. In DMSO solution, **3** oxidised to form a homodimeric disulphide. This species reacted quantitatively with 14-3-3σ using an equimolar ratio of reactants (both 100 μM) under non-reducing conditions, as shown by mass spectrometry analysis (Fig. 2A and S1†). Despite the *in vitro* requirement for non-reducing conditions, **3** is known to covalently modify proteins in the cellular environment, and this might be particularly prevalent in cells under oxidative stress.^18^ Disulphide exchange was unaffected by prior complexation of 14-3-3σ with a phospho-peptide mimicking the binding motif of p53. The corresponding 14-3-3σ mutant Cys38Ala was not modified – supporting the hypothesis of Cys38 covalent modification (Fig. S2†). This was expected because the only other Cys residue in the 14-3-3 sequence, Cys96, is not solvent exposed (based on the available structural data). Cys38 was previously shown to undergo disulphide exchange in a tethering screen.^24^ However, no effect on partner protein binding has previously been attributed to modification of the 14-3-3σ-unique Cys38 residue. Covalent imine tethering to Lys122 of 14-3-3 proteins has yielded stabilisers that are selective for specific 14-3-3 binding partners, but Lys122 is conserved across the 14-3-3 family and thus its modification would not constitute an isoform specific approach.^25,26^

To further profile the reactivity of Cys38, 14-3-3σ was incubated with the small sub-set of disulphide containing compounds **4–7**, again using 100 μM of both reactants, and analysed by mass spectrometry (Fig. 2B and S3A–D†). Cystamine (**4**), an endogenous disulphide-containing molecule that is structurally related to **3**, reacted quantitatively with the protein, whilst the non-natural di-*N*-propyl disulphide (**5**) and dithiopropionic acid (**6**) did not show any reactivity. This suggests that covalent protein modification at Cys38 is reliant on a basic amine in proximity to the electrophilic disulphide bond. Glutathione disulphide (**7**), a cell endogenous species, showed some reactivity, resulting in 8% ligation to the protein under these conditions. This is interesting because it suggests that Cys38 could play a broader chemical role in the cellular environment, and contribute to the unique activity profile of 14-3-3σ.

### WR-1065 stabilises 14-3-3σ PPIs

Two 14-3-3 partner protein binding motifs were compared in order to establish if **3** selectively influenced their binding to 14-3-3σ. The motifs for p53 (ref. 10) and for ERα^9^ were selected because of their different 14-3-3 binding profiles and our interest in them as potential oncology drug targets. Of the three possible binding motifs in p53, that around pT387 has been studied in most detail at the molecular level. It adopts a unique binding conformation with a turn local fold (Fig. 1B).^10^ This interaction is moderately stabilised by **1** (4×).^10^ By contrast, ERα has a characteristic ‘mode 3’ C-terminal binding conformation that binds with 48-fold greater affinity and is preferentially stabilised by **1** (34×).^9^

#### Fluorescence polarisation (FP)

**Fig. 3:**
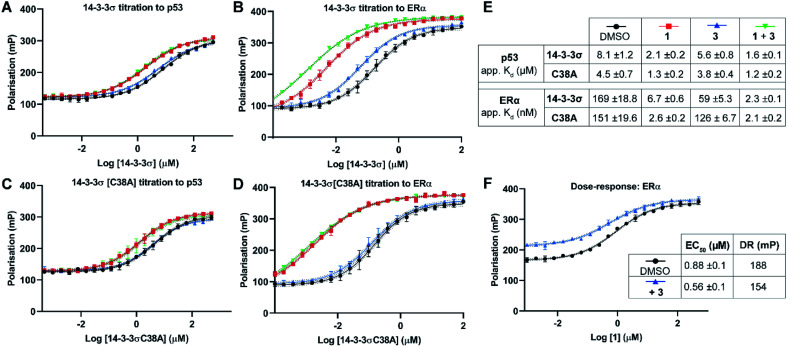
Fluorescence polarisation experiments show that **3** stabilises 14-3-3σ PPIs in a Cys38 dependent manner. (A–D) 14-3-3σ titrations to 10 nM fluorescently labelled phospho-peptide binding motifs in the presence of DMSO (control), **1** (p53: 1.0 mM; ERα: 0.01 mM), **3** (1.0 mM) or a combination of **1** and **3**. (E) Legend and table summarising apparent *K*_d_ values for each binding curve. (F) Dose–response experiment where **1** was titrated to 14-3-3σ (10 μM) alone or with **3** (10 μM). DR = dynamic range. Data points were recorded in triplicate and error is shown as the standard deviation from the mean. Data is representative of duplicate experiments – see Fig. S4† for data.

FP assays were used to determine if covalent modification of Cys38 by **3** influenced the binding affinity of partner protein binding motifs to 14-3-3σ. 14-3-3σ was titrated to a fixed concentration of fluorescently labelled phospho-peptide designed to mimic the respective 14-3-3σ binding motif. In the absence of **3**, 14-3-3σ bound to p53 and ERα phospho-peptides with apparent dissociation constant (*K*_d_) values of 8.1 ± 1.2 μM and 0.17 ± 0.02 μM respectively, in agreement with previous reports.^9,10^ In the presence of a molar excess of **3**, the affinity of 14-3-3σ binding to p53 was only enhanced by 1.4-fold (*K*_d_ → 5.6 ± 0.8 μM, Fig. 3A). However, there was a significant effect on the 14-3-3σ interaction with ERα whereby the affinity was enhanced by 2.8-fold (*K*_d_ → 59 ± 5.3 nM, Fig. 3B). Next, we investigated if **3** influenced the stabilising effect of **1** on these PPIs. In the presence of fixed concentrations of **1**, the apparent *K*_d_ for 14-3-3σ binding to ERα and p53 was lowered to 6.7 ± 0.6 nM (25×) and 2.1 ± 0.2 μM (4×) respectively (Fig. 3A and B). Both observations were again consistent with literature reports.^9,10^ Intriguingly, when **3** was used in combination with **1**, the stabilising effect on the 14-3-3σ interaction with ERα was further enhanced by 3-fold: apparent *K*_d_ 6.7 ± 0.6 nM → 2.3 ± 0.1 nM (Fig. 3B). However, the affinity of 14-3-3σ binding to p53 was again only slightly enhanced: apparent *K*_d_ 2.1 ± 0.2 μM → 1.6 ± 0.1 μM (1.3×, Fig. 3A). This data shows that **1** and **3** operate in a cooperative manner and that the effect is more pronounced for the 14-3-3σ–ERα PPI.

To confirm that **3** was exerting its effect *via* covalent modification of Cys38, the same experiments were performed using a 14-3-3σ Cys38Ala mutant construct (Fig. 3C and D). The apparent *K*_d_ values for 14-3-3σ Cys38Ala binding to both peptides alone, and in the presence of **1**, were broadly comparable with the wild-type construct. However, in all cases the effect of **3** was abrogated, thus supporting a covalent mode of action. The same observation was made using 14-3-3ζ, an isoform that does not have an analogous cysteine residue (Fig. S5†). This is significant because it suggests that covalent modification of Cys38 provides a strategy for selective targeting of the 14-3-3σ isoform.

Dose–response experiments further confirmed the specificity of the cooperative effect between **1** and **3** for the 14-3-3σ ERα PPI. Titration of **1** to fixed concentrations of 14-3-3σ and the respective fluorescently labelled ERα phospho-peptide gave an EC_50_ of 0.88 ± 0.1 μM (Fig. 3F). Upon the addition of **3** at an equimolar concentration to 14-3-3σ, this EC_50_ value was lowered relative to the control experiment (0.88 ± 0.1 μM → 0.56 ± 0.1 μM) (Fig. 3F). In addition, the dynamic range of the assay was reduced, thus confirming the independent and cooperative stabilising effects of **3** on this PPI. In the case of the 14-3-3σ–p53 PPI a reduced dynamic range was also observed in the presence of **3** (see Fig. S6†). However, **1** is not a potent stabiliser of this PPI and there was no detectable change in EC_50_ in the presence of **3**.

#### Isothermal titration calorimetry (ITC)

**Fig. 4:**
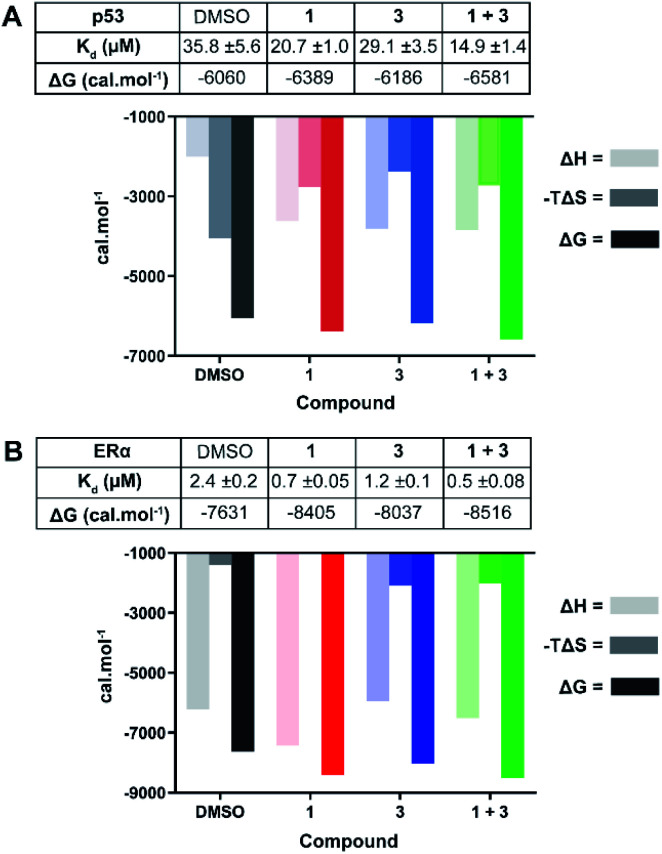
ITC experiments confirm the stabilising effect of **3** on the 14-3-3σ interactions with p53 (A) and ERα (B). (A) Unlabelled p53 phospho-peptide (1.0 mM) was titrated to 14-3-3σ (0.1 mM) in the presence of DMSO, **1** (1.0 mM), **3** (0.1 mM), or a combination of the two. (B) Unlabelled ERα phospho-peptide (0.5 mM) was titrated to 14-3-3σ (0.1 mM) in the presence of DMSO, **1** (0.1 mM), **3** (0.1 mM), or a combination of the two. See Fig. S7 and S8† for titration curves.

We used ITC to further investigate how **3** influenced the binding of the phospho-peptide motifs to 14-3-3σ. Unlabelled phospho-peptides were titrated to a fixed concentration of 14-3-3σ in the presence of **1**, **3**, or a combination of both compounds. The data obtained was compared to that for the interactions in the absence of the ligands. At an equimolar concentration relative to 14-3-3σ, **3** enhanced the affinity of both interactions in a manner that was consistent with the FP data. The *K*_d_ for the 14-3-3σ interaction with p53 was reduced only modestly (1.2 times) by addition of **3**: *K*_d_ 35.8 ± 5.6 μM → 29.1 ± 3.5 μM (Fig. 4A). This effect appears to be driven by a much increased enthalpic contribution to Δ*G* compared to the binary interaction that is predominantly driven by an increase in entropy, presumably due to a hydrophobic effect. In line with the FP data, **3** had a more pronounced effect on the interaction with ERα, enhancing the affinity by a factor of two: *K*_d_ 2.4 ± 0.2 μM → 1.2 ± 0.1 μM (Fig. 4B). In this case the effect appears to be driven by an increased entropic contribution to Δ*G*. These differing thermodynamic profiles indicate that **3** interacts differently with the respective binary complexes.

In agreement with the FP data, **1** also stabilised the 14-3-3 complex with the two peptides to different extents, the stabilisation being further enhanced upon the addition of **3**. In the case of p53, a molar excess of **1** relative to 14-3-3σ increased affinity by 1.7×, an effect that was further enhanced by 1.4× in the presence of **3** (Fig. 4A): *K*_d_ 35.8 ± 5.6 μM (DMSO) → 20.7 ± 1.0 μM (**1**) → 14.9 ± 1.4 μM (**1** + **3**). For ERα, addition of an equimolar concentration of **1** relative to 14-3-3σ enhanced the affinity by 3.4×. In this experiment, **3** enhanced this effect to the same degree as with p53, 1.4× (Fig. 4B): *K*_d_ 2.4 ± 0.2 μM (DMSO) → 0.7 ± 0.05 μM (**1**) → 0.5 ± 0.08 μM (**1** + **3**). The stabilisation offered by the addition of **3** to the 14-3-3σ : ERα : **1** ternary complex appears to be driven by a favourable enthalpic contribution without additional entropic costs, a hallmark of ligands that adopt several conformations.^27^ In other words, based on the ITC data, **3** may not fully order itself upon associating with the 14-3-3σ : ERα : **1** ternary complex, likely binding in a number of different and interconverting poses, each contributing favourable interactions to the enthalpy of binding. Overall, the ITC data provides further evidence for cooperative stabilisation of the 14-3-3σ PPIs by **1** and **3**.

### Structural basis for stabilisation of the 14-3-3σ–ERα PPI

In order to establish a structural rationale for the cooperative stabilising effect of **3** in combination with **1**, we determined the crystal structure of 14-3-3σ in complex with an ERα phospho-peptide (in the presence of which the effect was most pronounced) and ligands **1** and **3**.

Crystals of an ERα-8mer phospho-peptide mimicking the 14-3-3 binding motif (^588^AEGFPApTV^595^)^9^ in complex with 14-3-3σ were obtained at 4 °C within 3–5 days. Two 14-3-3σ : ERα crystals were separately soaked at 4 °C with: (i) **3** (40 mM, 2 h) and; (ii) an equimolar mixture of **1** and **3** (both 10 mM, overnight). The crystals were flash cooled in liquid nitrogen and exposed to X-rays. The 14-3-3σ : ERα crystal soaked with **3** gave a 1.19 Å resolution dataset and that soaked with **1** and **3** gave a 1.48 Å dataset (see Table S1† for diffraction data statistics). Initial phases for the structure factor amplitudes of the 14-3-3σ : ERα crystals soaked with **3** or **1** and **3** were computed by rigid-body refinement starting from the PDB ID 4JC3 and 4JDD models, respectively (these entries are crystal structures of human 14-3-3σ : ERα phospho-peptide and of a human 14-3-3σ : ERα : **1** ternary complex, respectively; see ESI† for further details on structure determination and refinement).^9^

**Fig. 5:**
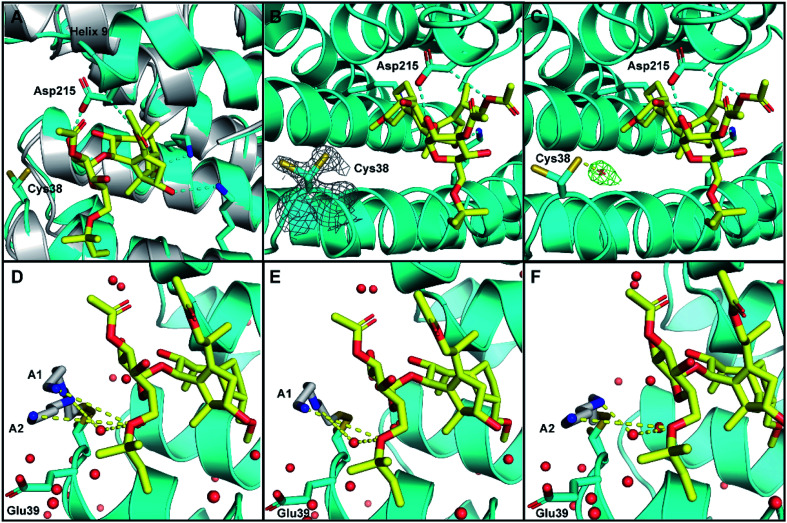
Crystal structure of human 14-3-3σ protein and ERα phospho-peptide alone (PDB ID: 7NIZ) and in complex with ligands **1** and **3** (PDB ID: 7NFW). (A) Overview of 14-3-3σ monomer complexed with ERα before soaking (grey) and after soaking (cyan) with **1** (yellow C atoms, red O atoms). (B and C) Electron density map 2Fo–Fc ((C); contoured as a grey mesh at 1*σ*) and residual electron density map Fo–Fc ((D); contoured as a green mesh at 2*σ*) around the side chain of Cys38 of 14-3-3σ (cyan). (D–F) Cys38 side chain, modified with **3** (grey), modelled in conformation A1 (panels (D and E), occA1 = 0.28) and A2 (panels (D and F), occA2 = 0.27) forming H-bond interactions *via* an ordered water molecule, and direct long range interactions with **1** (yellow dashed lines). The third Cys38 conformation B (occB = 0.45) points away from 14-3-3σ binding groove and is not shown.

As expected, in both structures the 14-3-3σ protein monomer accommodated one ERα phospho-peptide. In the crystal soaked with **3** alone no ordered density for the ligand was visible. In the crystal soaked with both **1** and **3**, one molecule of **1** was observed to nestle in proximity to the C-terminus of ERα, in a manner consistent with that previously observed (PDB ID 4JDD).^9^ Polar contacts between Asp215 of 14-3-3σ and **1** lead to a shift of helix 9 (aa 210–230) in a clamping motion towards the ligand (Fig. 5A). The data also revealed at least two conformations for the side chain of Cys38 (see the 2Fo–Fc electron density map, contoured at 1*σ* in Fig. 5B). Crucially, a positive peak in the Fo–Fc difference electron density map was found 2.0 Å away from the *S*_γ_ of the main conformer of the Cys38 side chain (see the Fo–Fc residual electron density map contoured at 2*σ* in Fig. 5C). This peak is compatible with a disulphide bond between Cys38 and the sulphur atom of **3**, confirming covalent modification of 14-3-3σ. Disappointingly, the rest of the ligand was largely absent from the maps, indicating disorder and partial occupancy of multiple poses.

To provide insight into how the predominant binding poses of **3** relate to that of **1** and ERα, the Cys38 side chain was first modelled in three conformations, fitting positive residual electron density. Two of these (conformations A1 and A2) point into the 14-3-3σ binding groove towards **1** and bear a disulphide bond to a distinct pose of **3**. For both conformations, one joint occupancy was refined for the Cys38 side chain and the corresponding pose of the covalently bound ligand **3**: occ_A1_ = 0.28; occ_A2_ = 0.27 (Fig. 5D). The third Cys38 conformation (occ_B_ = 0.45) points away from the binding groove and is free of ligand. Refinement statistics at the end of several cycles of iterative model building in Coot and refinement in autoBUSTER are gathered in Table S2.†

The A1 and A2 poses of **3** point towards the glycan ring of **1** and Glu39 of 14-3-3σ, the latter of which delineates the edge of the binding groove (Fig. 5D). Electrostatic interaction(s) between the positively charged amine(s) in **3** and the negatively charged acidic side chain of Glu39 may play a role in orientating **3** into this favourable position for disulphide bond formation with Cys38. This hypothesis is consistent with the mass spectrometry data that showed the requirement of a basic amine residue for covalent binding of disulphide compounds to 14-3-3σ (Fig. 2).

For both poses A1 and A2, the secondary amine of **3** appears to form polar contacts with an ordered water molecule that also coordinates to the oxygen of the allyl ether glycan substituent of **1** (Fig. 5E and F). This hydrogen bonding network is a highly plausible explanation for the biophysical data that suggests interaction between the two ligands. It is also possible that the terminal amine of **3** participates in a longer range attractive interaction with the same allyl ether substituent of **1** (*e.g.* ion-induced dipole interaction).

### WR-1065 and fusicoccin A kill senescent EJp53 cells in a cooperative and p53-specific manner

**Fig. 6:**
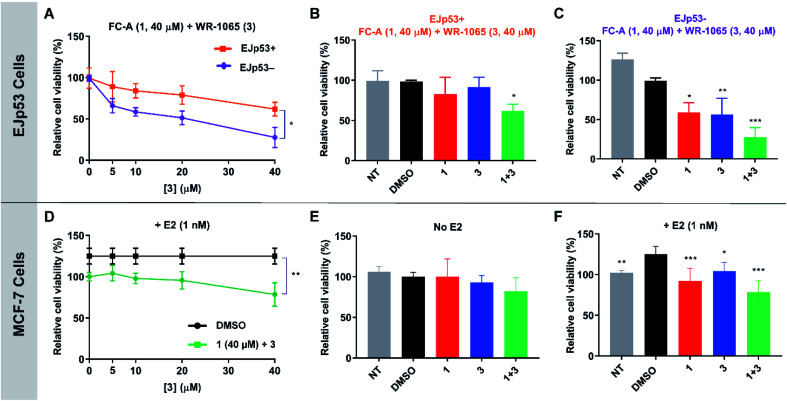
CTG assay data showing the cooperative and specific effects of **1** and **3** in EJp53 bladder carcinoma cells (A–C) and MCF-7 breast cancer cells (D–F). (A) EJp53 cells were treated with **1** (40 μM) and varying concentrations of **3** (0–40 μM). Metabolic activity was quantified after 72 hours. A *T*-test statistical analysis was performed. (B and C) Relative cell viability of EJp53+ and EJp53− cells treated with 40 μM of **1**, **3** or both. A two-way analysis of variance statistical analysis was performed. (D) MCF-7 cells were treated with **1** (40 μM) and varying concentrations of **3** (0–40 μM). Metabolic activity was quantified after 72 hours. A *T*-test statistical analysis was performed. (E and F) Relative cell viability of MCF-7 cells ± 1 nM E2 treated with 40 μM of **1**, **3** or both. A two-way analysis of variance statistical analysis was performed. NT: non-treated cells. All data were normalised to a DMSO treated control. Experiments were performed in technical triplicate and are representative of at least two independent repeats. Error shown is the standard deviation from the mean (*p* < 0.12 (ns), *p* < 0.033 (*), *p* < 0.002 (**), *p* < 0.001 (***)).

To validate the cooperative effect of **1** and **3** on p53 in a biological context, the compounds were tested against the EJp53 bladder carcinoma cell line.^28^ EJp53 has a tetracycline (TET)-off system for regulating the expression of p53.^29^ When TET is removed from the culture media (EJp53−), wild-type p53 is expressed and the cells enter a senescent state (Fig. S9†). When TET is retained in the culture media (EJp53+), p53 is not expressed and the cells proliferate. Thus, specific activity against EJp53− cells would be a strong indication of p53 activation that is consistent with stabilisation of the 14-3-3σ–p53 PPI. Both senescent and proliferating cells were separately treated with **1**, **3**, and with combinations of the two molecules, and metabolic activity was quantified after 72 hours using a CellTiter Glo (CTG) assay (Fig. 6A–C). In isolation, **1** and **3** both gave rise to a modest, but insignificant, decrease in cell viability of EJp53− senescent cells compared to the proliferating EJp53+ cells (Fig. S10†). However, when a combination of **1** (40 μM) and increasing concentrations of **3** were used, a significant dose-dependent cytotoxic effect was observed in EJp53− cells relative to the effect on EJp53+ cells (Fig. 6A). The IC_50_ of the combination treatment in EJp53− cells was calculated as 15.1 ± 2.1 μM compared to that in EJp53+ cells of 77.6 ± 38.2 μM. At the highest concentration point used (40 μM of **1** and **3**) cell viability was reduced to 28% relative to a DMSO control (Fig. 6C). In contrast, the viability of the EJp53+ proliferating cells was reduced to a much lesser extent (64%, Fig. 6B). The same effect was observed when the concentration of **3** was fixed at 40 μM, and the concentration of **1** was varied (Fig. S10†). These data are a strong indication that **1** and **3** have a cooperative cytotoxic effect against senescent EJp53 cells in a manner that is dependent on p53 expression.

To further confirm that the effect of the two ligands was specific to p53, the same experiments were performed in the EJp21 cell line.^30^ EJp21 cells are p53-null but can undergo senescence by activation of p21 *via* a TET-off system (Fig. S9†). Neither ligand by itself, or any combination of the two, had any cytotoxic effect on proliferating or senescent cells (Fig. S11†).

Together, these observations demonstrate the cellular relevance of the cooperative behaviour of **1** and **3.** In combination, the compounds effectively reduce the viability of senescent EJp53 cells in a p53-specific manner, which is a strong indication of an enhancement of p53 activity. Thus, a cell cycle arrest response is turned into cell death, consistent with previous reports showing that senescence can be converted into apoptosis by increasing p53 activity.^28^ These data cannot explicitly attribute this to stabilisation of the 14-3-3σ–p53 PPI, but the results are remarkably consistent with the effect that is to be expected based on the *in vitro* biophysical data.

### WR-1065 and fusicoccin A cooperatively attenuate estradiol-induced MCF-7 cell proliferation

Next, the cooperative effect of **1** and **3** against the MCF-7 breast cancer cell line was investigated. MCF-7 cell proliferation is regulated by ERα dimerisation which leads to its enhanced interaction with chromatin and the expression of pro-survival target genes. ERα dimerisation is promoted by binding of 17β-estradiol (E2), which increases MCF-7 cell proliferation. It was previously shown that this effect is attenuated by **1**, which prevents ERα dimerisation by stabilising its interaction with 14-3-3σ.^9^

A CTG assay was used to establish if **1** and **3** exhibited a specific effect against E2-induced MCF-7 cell growth. Cells were pre-treated separately with **1** or **3**, or a combination of the two, for one hour, before the addition of E2 or DMSO as a control. Metabolic activity was quantified after 72 hours. Treatment with 1 nM E2 resulted in a 25% increase in cell viability compared to non-treated cells. This effect was attenuated by the addition of **1** and **3** (Fig. S12†), but again a combination of the two resulted in the most significant effect (Fig. 6D), although IC_50_ values could not be calculated. At the highest concentration point used (40 μM of **1** and **3**), the combination treatment resulted in MCF-7 cell viability being reduced to 78% compared to **1** (92%) and **3** (104%) alone (Fig. 6F). Importantly, this effect was only observed in MCF-7 cells treated with E2 (Fig. 6E and S13†). Therefore, this is a strong indication that the cytotoxic activity of **1** and **3** is closely linked to antagonism of ERα dimerisation. As with EJp53, these data cannot explicitly prove that this results from stabilisation of the ERα–14-3-3σ PPI in MCF-7 cells, but they do provide compelling evidence in support of that hypothesis and further exemplification of cooperativity between **1** and **3**.

## Conclusions

Here, we report that covalent modification of 14-3-3σ Cys38 by the disulphide of WR-1065 (**3**) leads to isoform-specific stabilisation of two 14-3-3σ PPIs *in vitro*. Achieving isoform specificity is a major challenge that must be overcome for the maturation of 14-3-3 drug discovery programmes. Our findings demonstrate a novel strategy that can be further exploited in the development of 14-3-3σ specific stabilisers, and also for harnessing the inherent potency of covalent modulators. The data also indicate that *in vitro***3** preferentially stabilises the 14-3-3σ interaction with ERα, which binds 14-3-3σ *via* a C-terminal ‘mode 3’ phosphorylated binding motif, compared to p53 which adopts a unique turn conformation. In the absence of structures of ternary complexes containing **3** alone, the structural basis for this is not yet clear. However, the data does highlight the potential for achieving PPI selectivity in addition to isoform selectivity.

The cooperative behaviour of **1** and **3** reveals a fascinating stabilisation mechanism that is distinct from the only other example of cooperative dual ligand stabilisation of 14-3-3 PPIs.^16^ In that report, a supramolecular arginine mimetic was shown to act cooperatively with **1** to stabilise the 14-3-3ζ interaction with ERα.^16^ Although highly significant, that example was limited in terms of the physiochemical properties of the supramolecular ligand and scope for achieving isoform specificity. Here, the cellular relevance of the cooperative effect between **1** and **3** has been demonstrated in two cancer cell lines. Furthermore, our 14-3-3σ : ERα : **1** : **3** crystal structure provides a platform for structure-based design of more potent, drug-like and synthetically tractable analogues of **3**. It also highlights the potential impact of covalent hybrids of **3** and **1**, or other known 14-3-3 stabilisers that occupy a similar pocket. We envisage that the covalent and cooperative stabilisation mechanism revealed by this study will underpin the development of selective and efficacious 14-3-3σ stabilisers that can be exploited in a range of therapeutic areas such as oncology and neurodegeneration.

## Experimental

Full experimental details can be found in the ESI.†

## Data availability

Crystallographic data for this work has been deposited in the Protein Data Bank under 7NIZ and 7NFW and can be obtained from https://www.rcsb.org/. All other experimental data are available from the authors on request.

## Author contributions

MF, JAW, SYC, JB, PR and AM conducted the experimental work and edited the manuscript. MF, SM, PR and RGD analysed the data and wrote the manuscript. RGD conceptualised the study.

## Conflicts of interest

There are no conflicts to declare.

## Supplementary Material

SC-012-D1SC02120F-s001
