# Supplementary material for: Cooperative stabilisation of 14-3-3σ protein–protein interactions via covalent protein modification
Source: Chem Sci. 2021 Sep 6;12(39):12985–92. doi: 10.1039/d1sc02120f (PMC8513901; doi:10.1039/d1sc02120f)
Supplement: SC-012-D1SC02120F-s001 [file SC-012-D1SC02120F-s001.pdf]

Electronic Supplementary Material (ESI) for Chemical Science.
This journal is © The Royal Society of Chemistry 2021

Cooperative Stabilisation of 14-3-3σ Protein-Protein Interactions via Covalent Protein Modification

Marta Falcicchio[a,b], Jake A. Ward[a,c], Sara Y. Chothia[a,b], Jaswir Basran[d], Alisha Mohindra[a,b], Salvador Macip[c,e*], Pietro Roversi[a,d,f*], Richard G. Doveston[a,b*]

a. Leicester Institute for Structural and Chemical Biology, University of Leicester, University Road, Leicester, LE1 7HB, UK.

b. School of Chemistry, University of Leicester, University Road, Leicester, UK, LE1 7RH, UK.

c. Mechanisms of Cancer and Aging Laboratory, Department of Molecular and Cell Biology, University of Leicester, University Road, Leicester, LE1 7RH, UK.

d. Department of Molecular and Cell Biology, Henry Wellcome Building, University of Leicester, University Road, Leicester, LE1 7HB, UK.

e. FoodLab, Faculty of Health Sciences, Universitat Oberta de Catalunya, Barcelona, Spain.

f. Institute of Agricultural Biology and Biotechnology, IBBA-CNR Unit of Milano, via Bassini 15, I-20133, Milan, Italy.

Supporting Information

Contents

Supporting Figures…… 2-20

Supporting Tables…… 10-11

Experimental Procedures…… 21-24
Protein Expression…… 21
Phospho-peptide Information…… 22
Mass Spectrometry Experiments…… 22
Fluorescence Polarisation Experiments…… 22
Isothermal Titration Calorimetry Experiments…… 22
Crystallography…… 23
Cell lines and treatment…… 23
Detection of senescence-associated β-galactosidase (SA-b-gal)…… 23
CellTiter-Glo Viability Assay…… 24
Statistical Analysis…… 24
References…… 24

Supporting Figures

Mass Spectrometry Data (Figures S1-S3)

14-3-3σ
Calculated Molecular Weight: 31145.69 (with loss of N-terminal Met = 31014.49)
Observed Mass: 31014.85

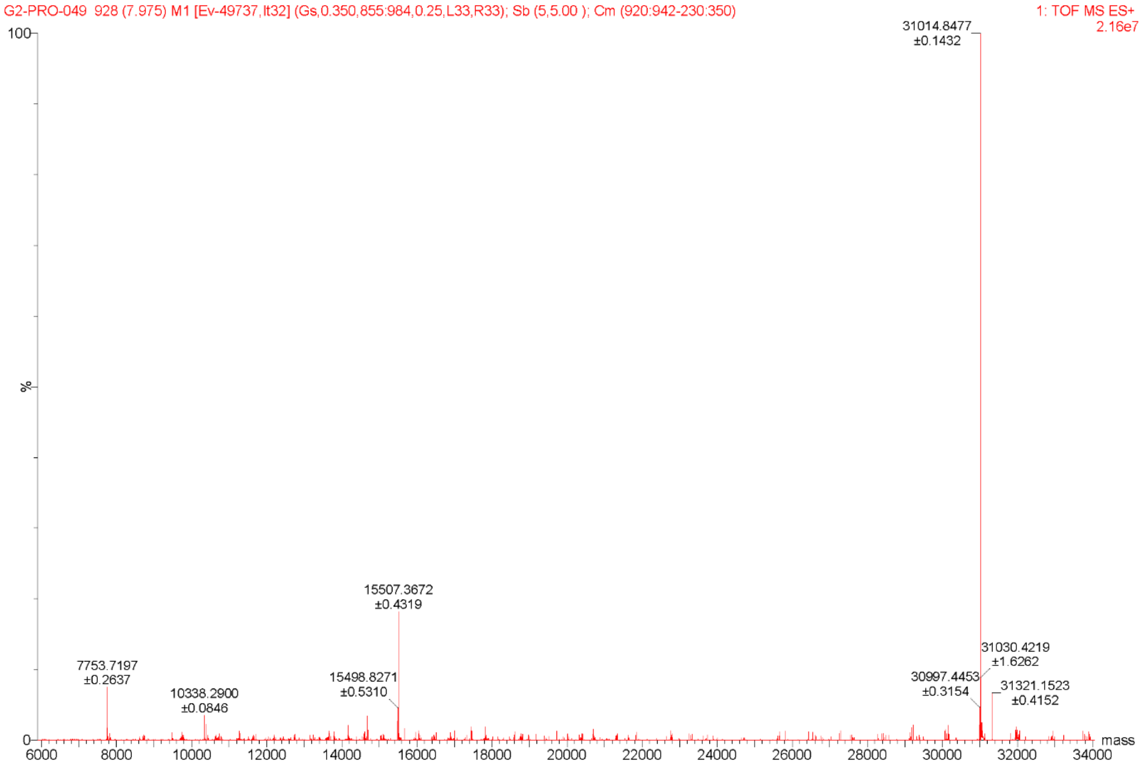

14-3-3σ + 3 (Mw = 134.24)
Modified Mass: 31146.73 (with loss of N-terminal Met)
Observed Mass: 31147.37

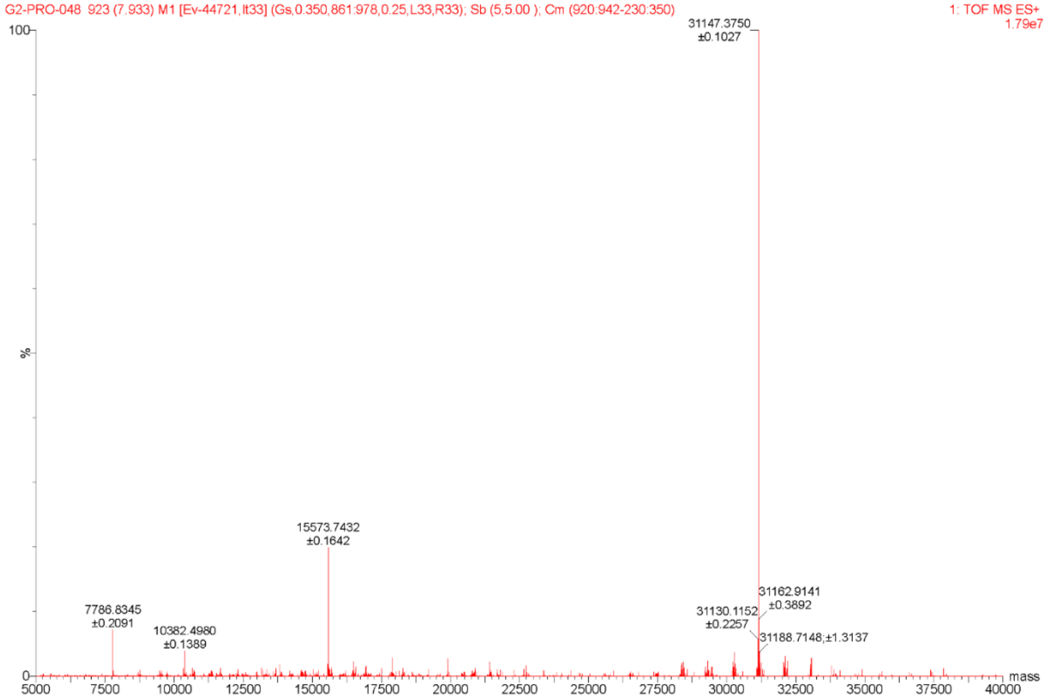

Figure S1. Deconvoluted mass spectra of 14-3-3σ (top) and 14-3-3σ after incubation with **3** (bottom).

14-3-3σ[C38A]
Calculated Molecular Weight: 31113.63 (with loss of N-terminal Met = 30982.43)
Observed Mass: 30982.16

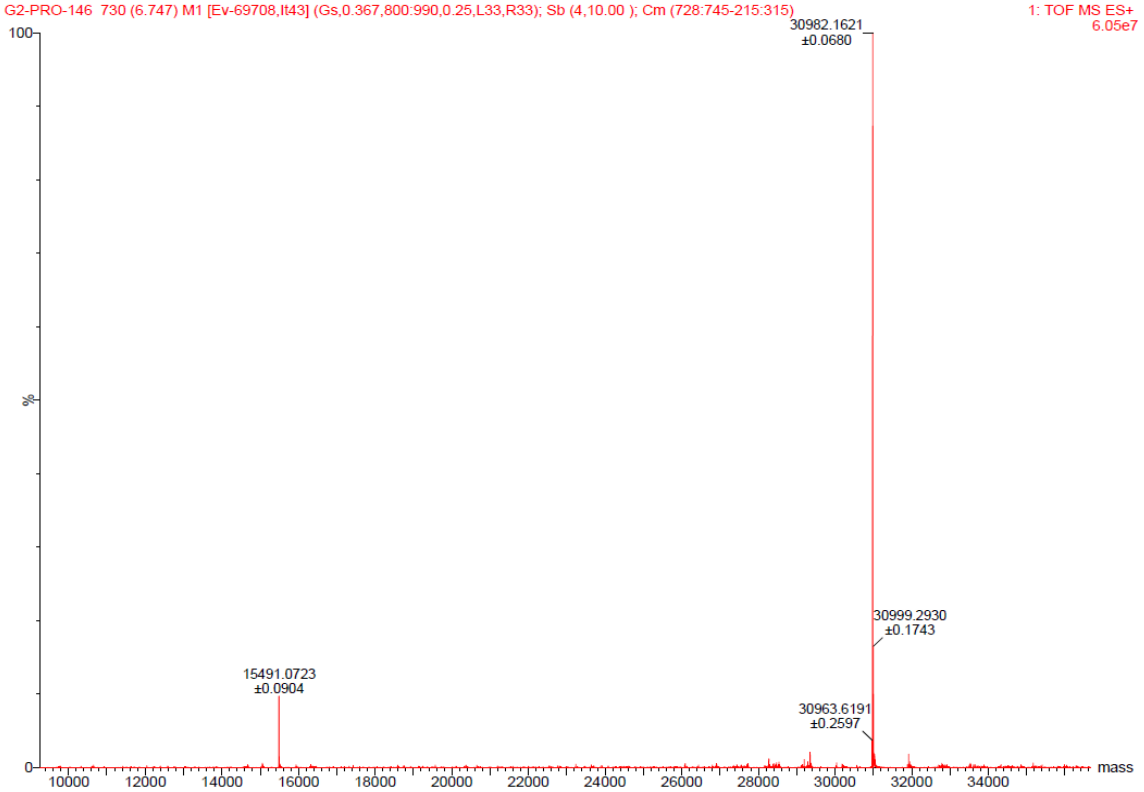

14-3-3σ[C38A] + 3 (Mw = 134.24)
Modified Mass: 31123.67 (with loss of N-terminal Met)
Observed Mass: 30982.25 (consistent with unmodified protein)

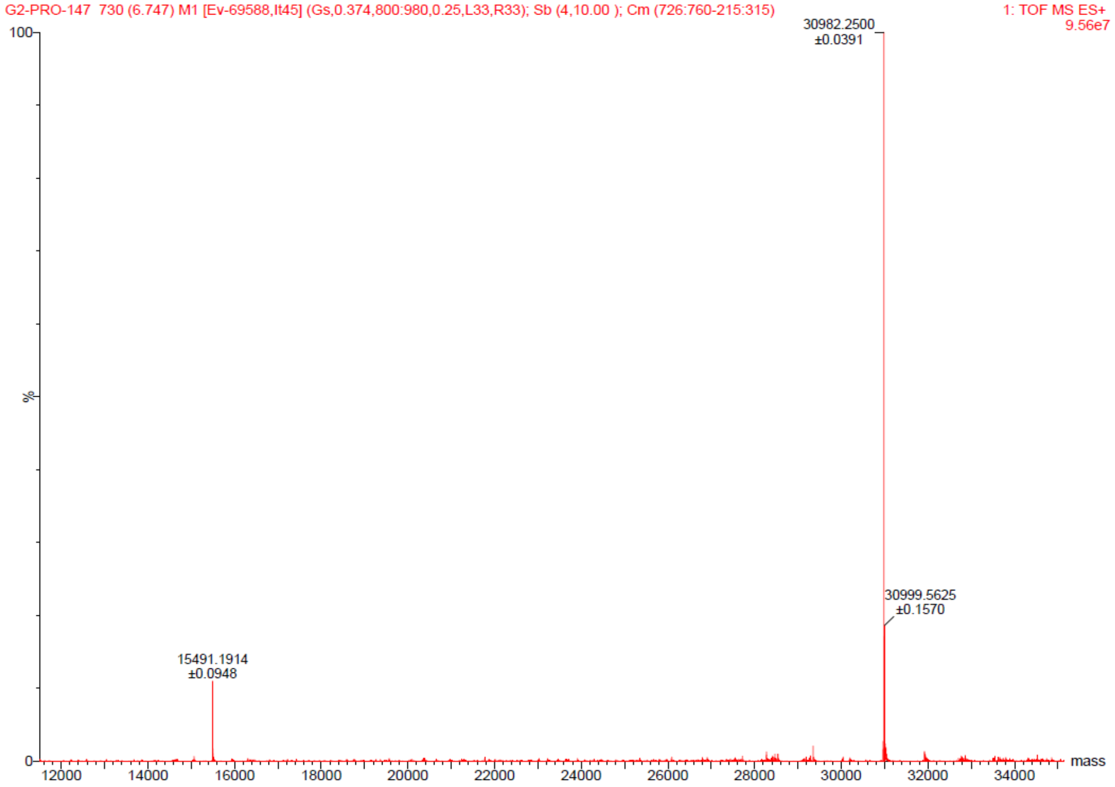

Figure S2. Deconvoluted mass spectra of 14-3-3σ[C38A] (top) and 14-3-3σ[C38A] after incubation with **3** (bottom).

A. 14-3-3σ + 4 (Mw of disulphide = 152.27)

Modified Mass: 31089.64 (with loss of N-terminal Met)

Observed Mass: 31089.81

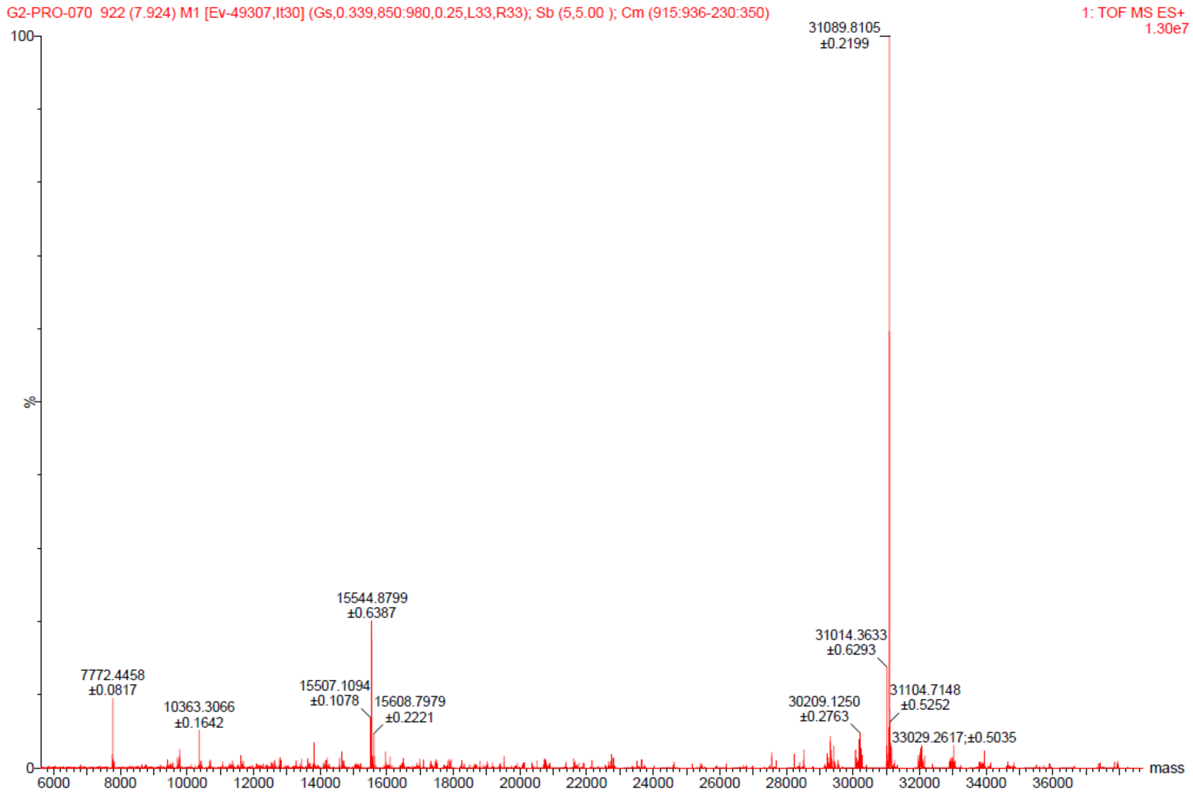

B. 14-3-3σ + 5 (Mw of disulphide = 150.30)

Modified Mass: 31088.65 (with loss of N-terminal Met)

Observed Mass: 31014.25 (consistent with unmodified protein)

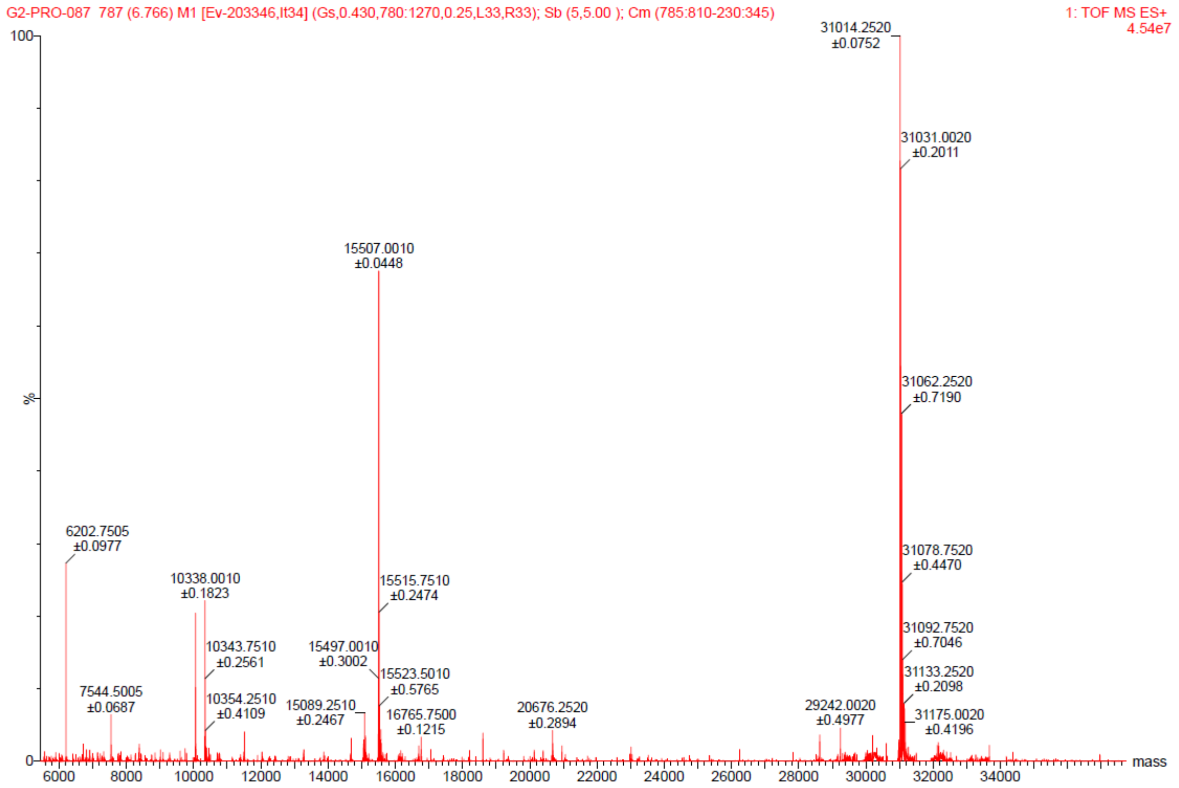

C. 14-3-3σ + 6 (Mw of disulphide = 210.27)

Modified Mass: 31120.63 (with loss of N-terminal Met)

Observed Mass: 31015.25 (consistent with unmodified protein)

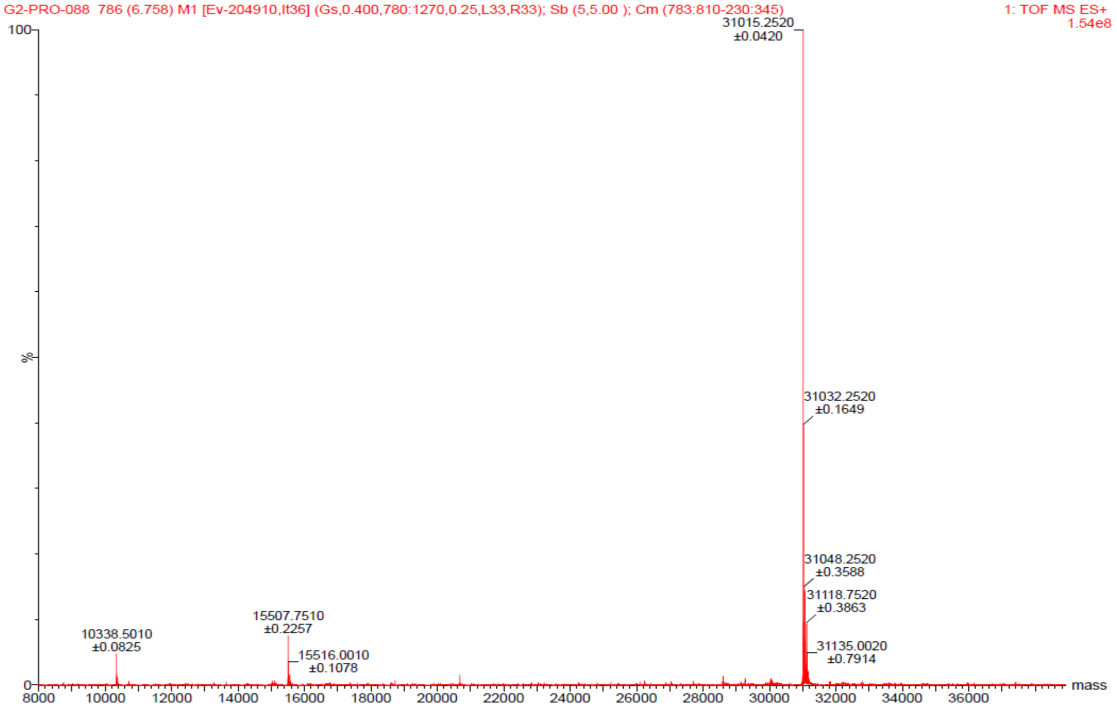

D. 14-3-3σ + 7 (Mw of disulphide = 656.59)

Modified Mass: 31321.81 (with loss of N-terminal Met)

Observed Mass: 31015.25 (100% - consistent with unmodified protein); 31320.95 (8%)

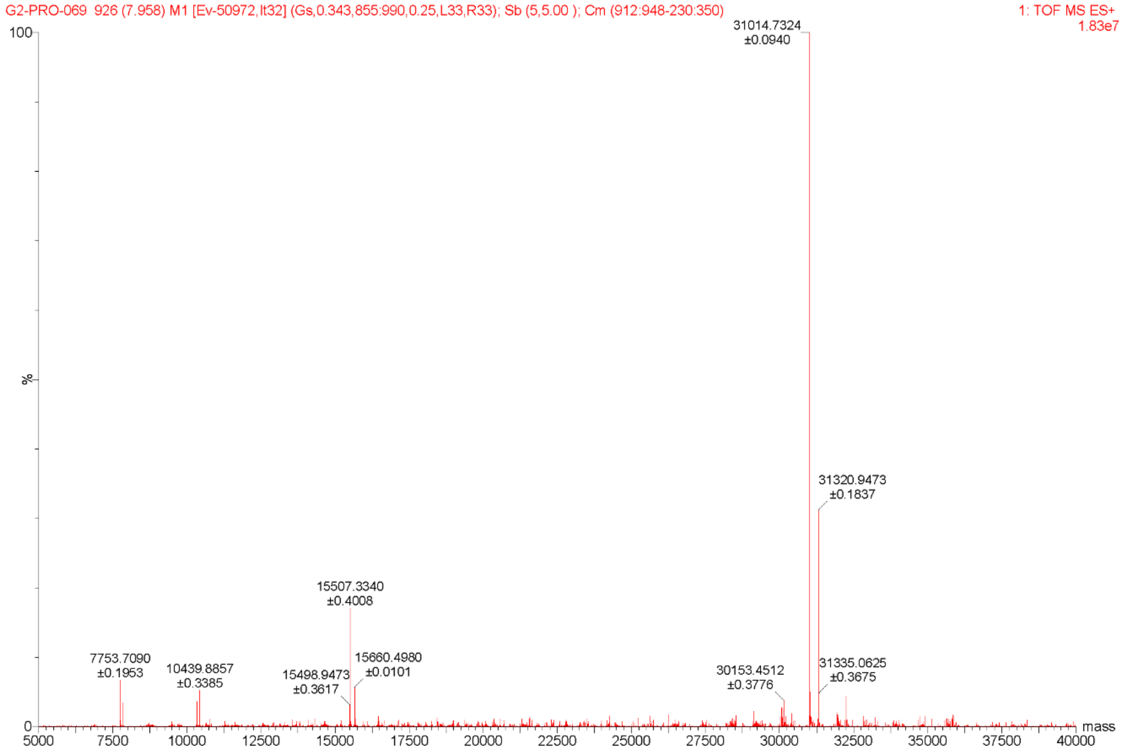

Figure S3. A-D: Deconvoluted mass spectra of 14-3-3σ after incubation with **4**-**7**.

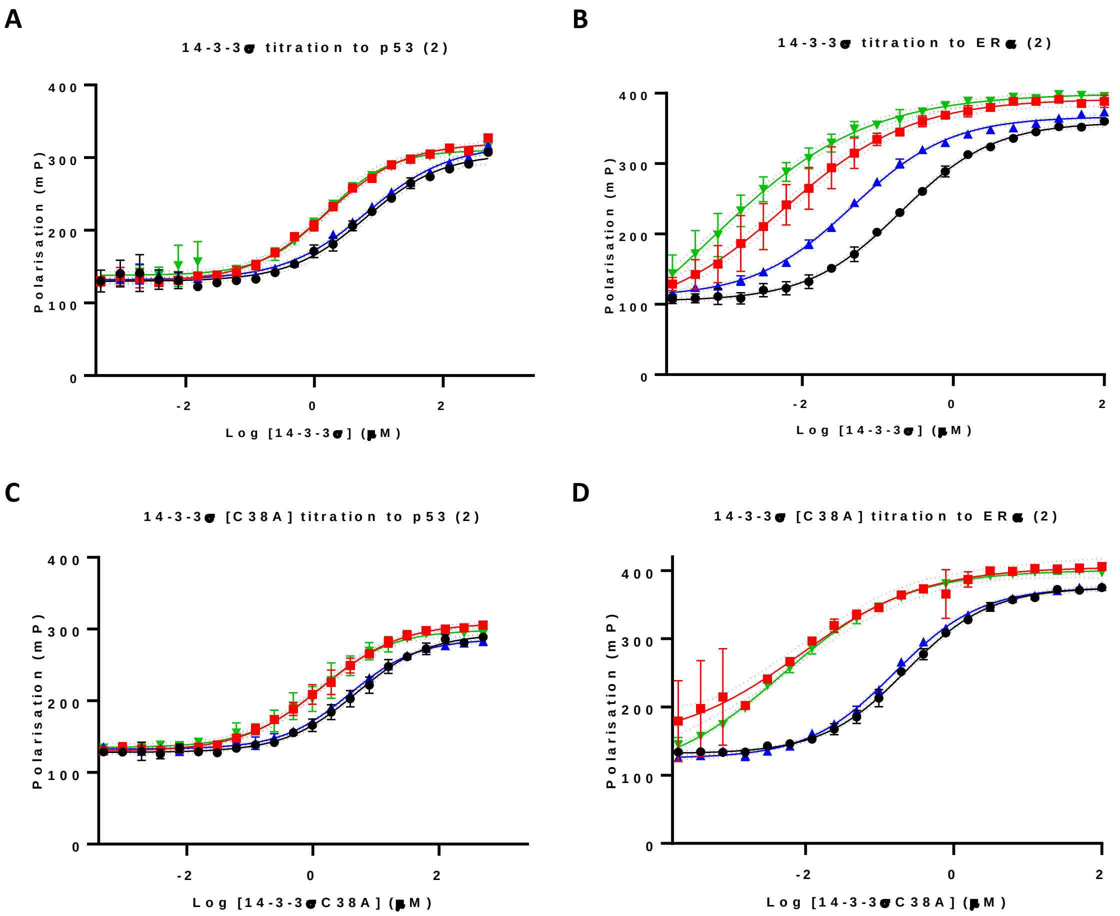

E

		DMSO	1	3	1+3
**p53** app. K_d (µM)	14-3-3σ	6.3 ±1.5	1.6 ±0.2	6.5 ±1.0	1.5 ±0.3
	C38A	4.8 ±0.7	1.4 ±0.2	3.7 ±0.3	1.3 ±0.2
**ERα** app. K_d (nM)	14-3-3σ	204 ±16.6	5.8 ±3.7	45 ±3.7	0.62 ±1.5
	C38A	228 ±17.6	8.3 ±9.5	158 ±11.0	4.8 ±1.0

F

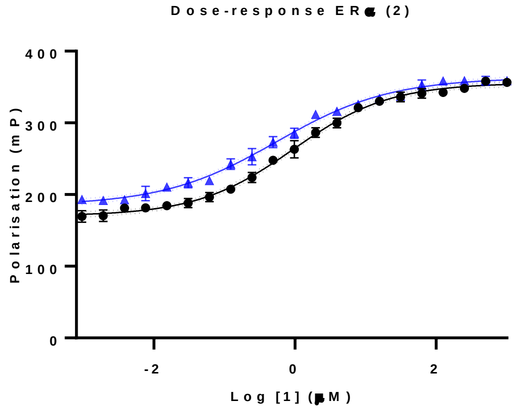

	EC_{50} (µM)	DR (mP)
DMSO	0.93 ±0.1	185
+3	0.54 ±0.1	178

Figure S4. Duplicate FP data in support of Figure 3. **A-D:** 14-3-3σ and 14-3-3[C38A] (p53: 0.5 mM; ERα: 0.1 mM) titrations to 10 nM fluorescently labelled phospho-peptide binding motifs in the presence of DMSO (control), **1** (p53: 1.0 mM; ERα: 0.01 mM), **3** (1.0 mM) or a combination of **1** and **3**. **E**: Legend and table summarising EC_{50} values for each binding curve. **F**: Dose-response experiment where **1** was titrated to 14-3-3σ (10 µM) alone or with **3** (10 µM). DR = dynamic range. Data points were recorded in triplicate and error is show as the standard deviation from the mean.

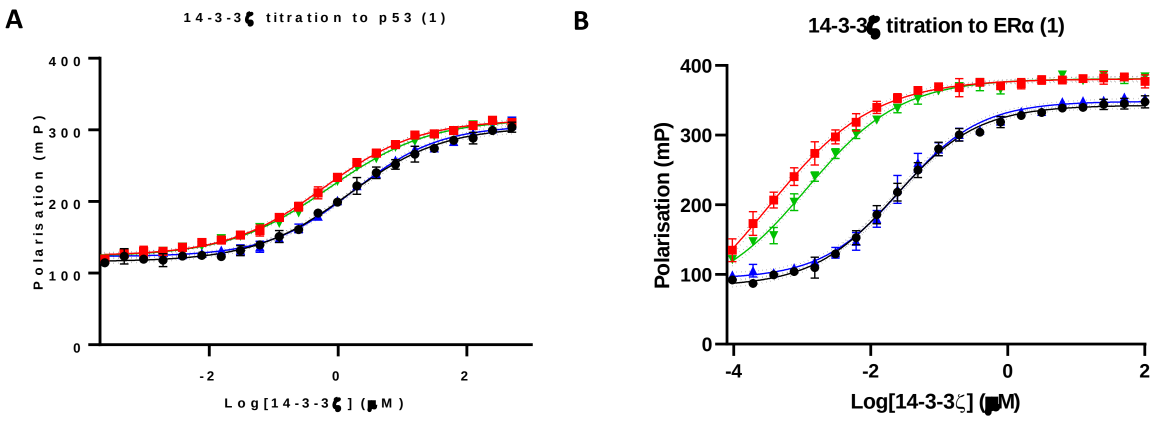

C

	DMSO	1	3	1+3
**p53** app. K_d (μM)	1.5 ±0.2	0.6 ±0.1	1.7 ±0.2	0.7 ±0.1
**ERα** app. K_d (nM)	227 ±24.9	3.1 ±2.3	274 ±33.9	11 ±3.0

Figure S5: FP data for 14-3-3ζ titration to p53 and ERα peptides. **A-B:** Titration of 14-3-3ζ (0.5 mM) to TAMRA-p53-CTD and TAMRA-ERα peptides (10 nM) in presence of DMSO (control); **1** (p53: 1 mM; Erα: 0.01 mM); **3** (1.0 mM); and a combination of **1** + **3.** **C:** Legend and table summarising EC_{50} values for each binding curve. DMSO was used at 1% v/v to balance the absence of the second ligand and 2% v/v for the control. DR = dynamic range. Data points were recorded in triplicate and error is show as the standard deviation from the mean.

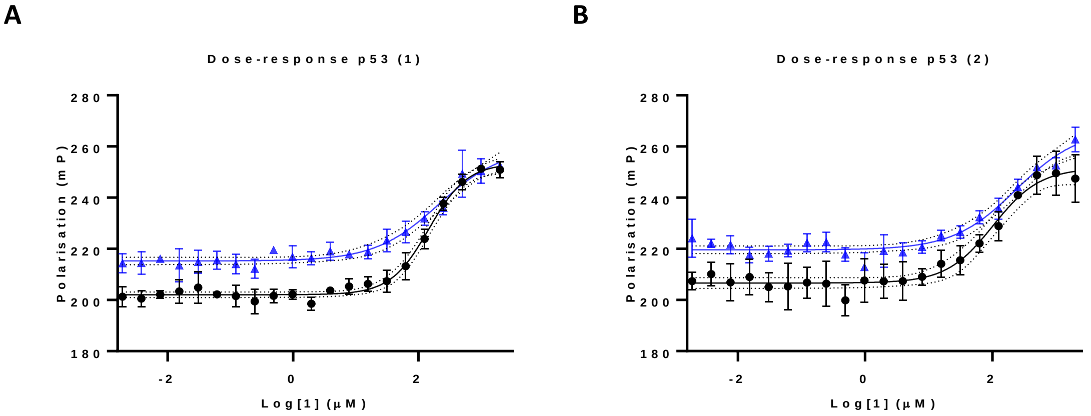

C

	Replicate	EC_{50} (μM)	DR (mP)
DMSO	1	146 ±43	202
	2	102 ±43	215
+3	1	210 ±144	206
	2	236 ±165	219

Figure S6: FP Data of FC-A dose-response to p53. **A-B:** Replicate 1 and 2 of dose-response experiment where **1** was titrated to 14-3-3σ (0.01 mM) alone or with **3** (0.01 mM). **C:** Legend and table summarising EC_{50} values for each binding curve. DR = dynamic range. Data points were recorded in triplicate and error is show as the standard deviation from the mean.

A

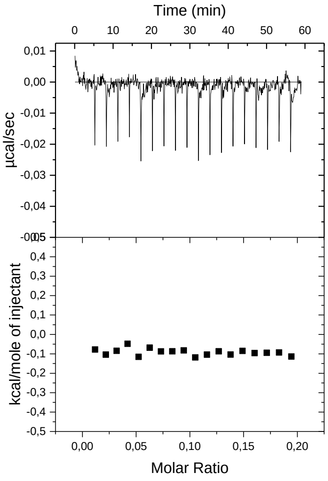

p53 (1mM)

B

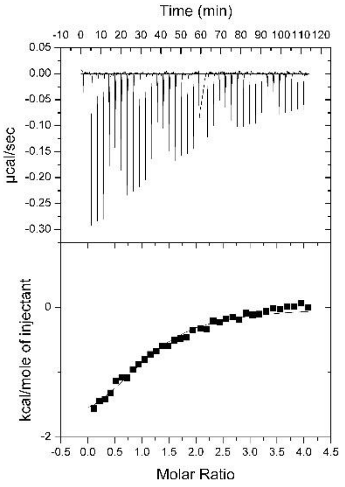

DMSO (Control)

N = 1.18 ± 0.0520 Sites

Kd = 35.84 ± 5.60 µM

ΔH = -2007 ± 125.4 cal/mol

ΔS = 13.6 (cal/mol)/deg

C

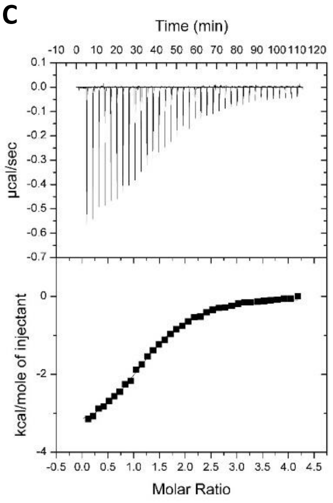

FC-A (1 mM)

N = 1.34 ± 0.0124 Sites

Kd = 20.66 ± 1.00 µM

ΔH = -3621 ± 46.54 cal/mol

ΔS = 9.29 (cal/mol)/deg

D

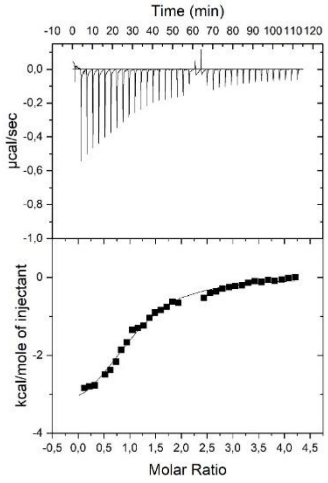

WR-1065 (0.1mM)

N = 1.12 ± 0.0333 Sites

Kd = 29.1 ± 3.52 µM

ΔH = -3814 ± 163.9 cal/mol

ΔS = 7.96 (cal/mol)/deg

E

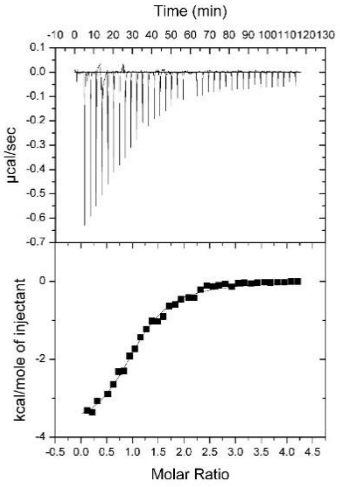

WR-1065 (0.1mM) + FC-A (1 mM)

N = 1.09 ± 0.0196 Sites

Kd = 14.93 ± 1.44 µM

ΔH = -3852 ± 94.65 cal/mol

ΔS = 9.16 (cal/mol)/deg

Figure S7: ITC Isotherms for p53 binding to 14-3-3σ in support of Figure 4. **A:** Unlabelled p53-CTD 15mer peptide (1.0 mM syringe concentration) was titrated into buffer and to 14-3-3σ (0.1 mM) in the presence of **B:** DMSO (control); **C: 1** (1 mM) **D: 3** (0.1 mM); **E: 1** + **3**. DMSO was used at 1% v/v to balance the absence of the second ligand and 2% v/v for the control.

A **B** **C**

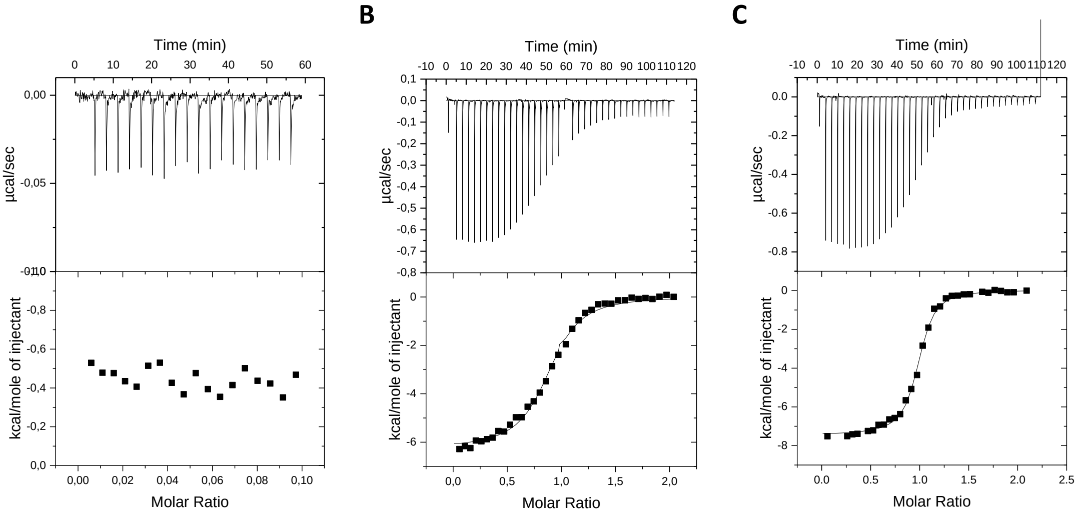

ERα (0.5mM)

DMSO (Control)

N = 0.893 ± 0.0073 Sites

Kd = 2.36 ± 0.22 µM

ΔH = -6224 ± 67.41 cal/mol

ΔS = 4.87 (cal/mol)/deg

FC-A (0.1 mM)

N = 0.972 ± 0.0038 Sites

Kd = 0.65 ± 0.05 µM

ΔH = -7429 ± 51.89 cal/mol

ΔS = 3.38 (cal/mol)/deg

D **E**

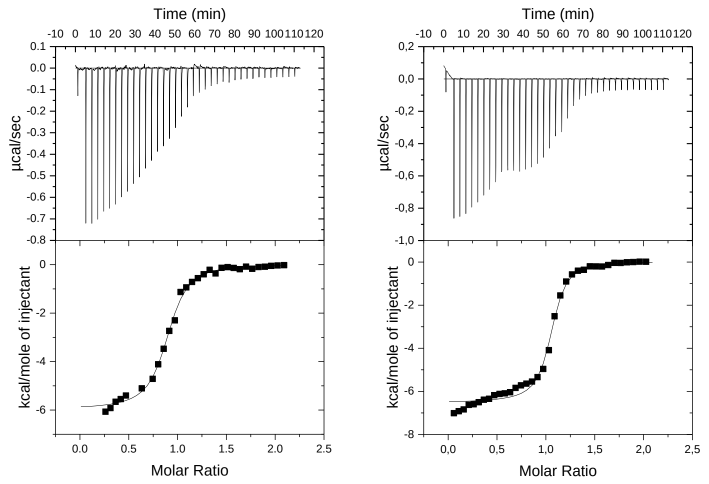

WR-1065 (0.1mM)

N = 0.889 ± 0.0054 Sites

Kd = 1.15 ± 0.11 µM

ΔH = -5942 ± 66.69 cal/mol

ΔS = 7.25 (cal/mol)/deg

WR-1065 (0.1mM) + FC-A (0.1 mM)

N = 1.04 ± 0.0066 Sites

Kd = 0.51 ± 0.08 µM

ΔH = -6508 ± 65.02 cal/mol

ΔS = 6.95 (cal/mol)/deg

Figure S8: ITC Isotherms for ERα binding to 14-3-3σ in support of Figure 4. **A**: Unlabelled ERα-15mer peptide (0.5 mM syringe concentration) was titrated into buffer and to 14-3-3σ (0.1 mM) in the presence of **B:** DMSO (control); **C: 1** (0.1 mM); **D: 3** (0.1 mM); **E: 1** + **3**. DMSO was used at 1% v/v to balance the absence of the second ligand and 2% v/v for the control.

Table S1. Diffraction data statistics. Crystal X-ray diffraction data collection parameters and data processing statistics for 14-3-3σ/Erα-8mer and 14-3-3σ/Erα-8mer/FC-A/WR-1065 complexes. Values in parentheses refer to the highest resolution shell.

**PDB ID**	**7NFW**	**7NIZ**
Structure contents	14-3-3σ/Erα-8mer	14-3-3σ/Erα-8mer/FC-A/WR-1065
Wavelength (Å)	0.99990	0.99990
Det. Dist. (mm)	220.7	220.7
Photon flux (10^{12}photons/s)	2.76	2.79
Transmission (%)	100	100
Number of images	3,600	3,600
Oscillation range (°)	0.10	0.10
Exposure time (s)	0.020	0.020
Space Group	$C\ 2\ 2\ 2_1$	$C\ 2\ 2\ 2_1$
Cell edges: a, b, c (Å)	82.007, 112.003, 62.511	84.208, 110.938, 62.589
Resolution Range (Å)	66.16-1.19 (1.29-1.19)	67.07-1.48 (1.64-1.48)
R_{merge}	0.262 (3.413)	0.499 (5.126)
R_{means}	0.273 (3.641)	0.520 (5.331)
Observations	903,468 (30,119)	430,375 (21,528)
Unique observations	75,691 (3,784)	33,462 (1,673)
Avarage I/σ(I)	10.2 (1.7)	7.3 (1.7)
Completeness	82.2 (21.4)	68.0 (12.8)
Multiplicity	11.9 (8.0)	12.9 (12.9)
$CC_{1/2}$	0.994 (0.221)	0.983 (0.131)

Table S2. Refinement statistics. Crystal structures refinement statistics for 14-3-3σ/Erα-8mer and 14-3-3σ/Erα-8mer/FC-A/WR-1065 complexes. Values in parentheses refer to the highest resolution shell.

**PDB ID**	**7NFW**	**7NIZ**
Structure contents	14-3-3σ/Erα-8mer	14-3-3σ/Erα-8mer/FC-A/WR-1065
Space Group (Z)	C 2 2 21	C 2 2 21
Resolution range (Å)	1.19-66.17	1.48-67.07
Reflex.s working set	21.03	21.08
Refelx.s free set	22.62	23.94
R,Rfree	0.2103/0.2262	0.2107/0.2394
Rmsdbonds (Å)	0.08	0.08
Rmsdangles (°)	0.9	0.9
Ramachandran fav. (%)	97.25	97.25
Ramachandran allow. (%)	2.29	2.75
Ramachandran outliers (%)	0.46	0.00
〈B〉prot (〈B〉wat) (Å2)	17.03	25.35

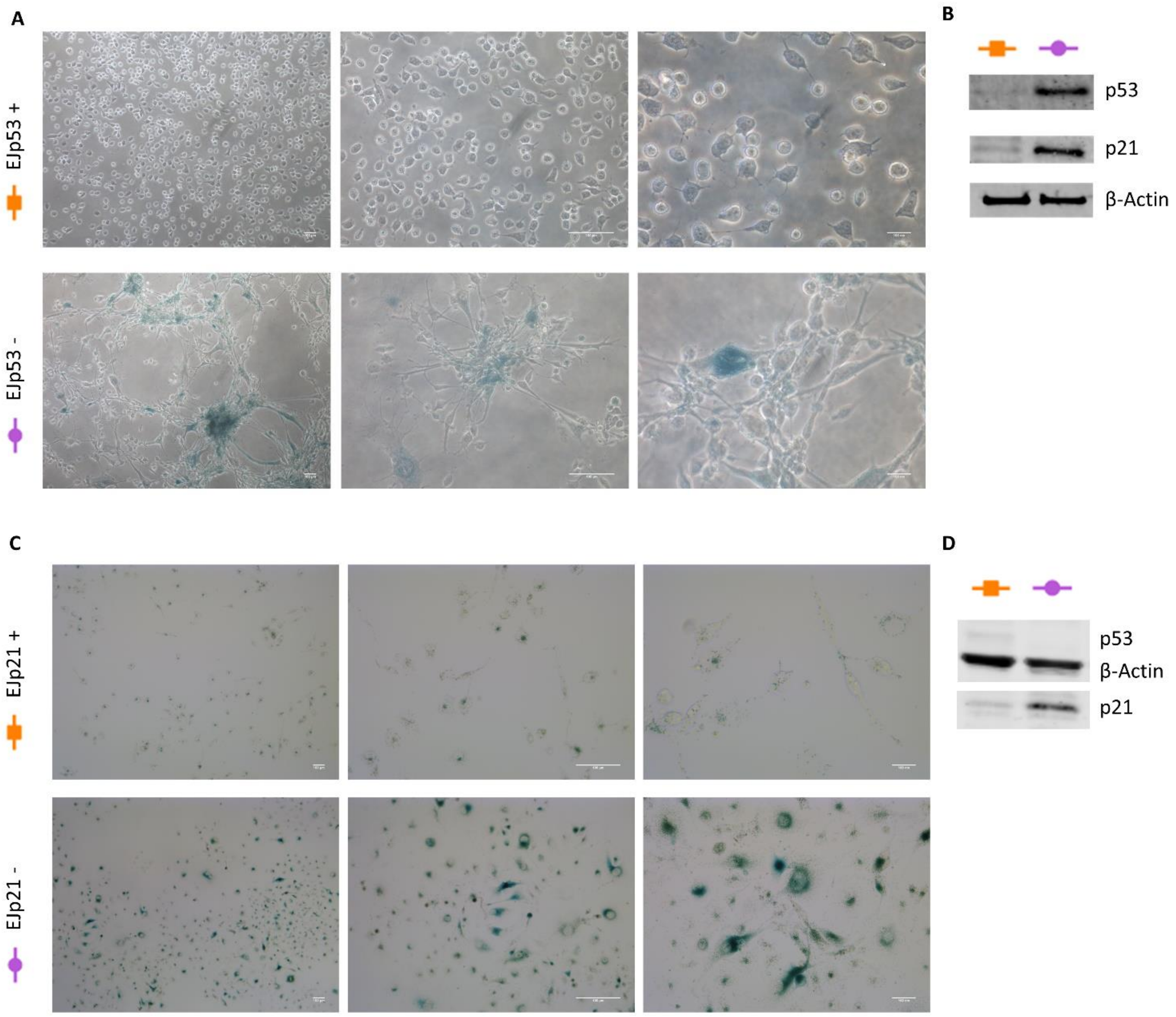

S9. Markers of senescence in EJp53 (A-B) and EJp21 cells (C-D). A: Senescence-associated beta-galactosidase (SA-β-Gal) staining of EJp53 cells uninduced (EJp53+) and 6 days after TET removal to induce senescence (EJp53–). Blue staining and morphological changes are indicative of senescence. **B**: Western Blot analysis showing p53, p21 and β-Actin (control) levels in lysates of EJp53 after 6 days from the removal of TET from the cells and with TET. **C**: Senescence-associated beta-galactosidase (SA-β-Gal) staining of EJp21 cells uninduced (EJp21+) and 6 days after TET removal to induce senescence (EJp21–). Blue staining and morphological changes are indicative of senescence. **D**: Western Blot analysis showing p53, p21 and β-Actin (control) levels in lysates of EJp53 after 6 days from the removal of TET from the cells and with TET.

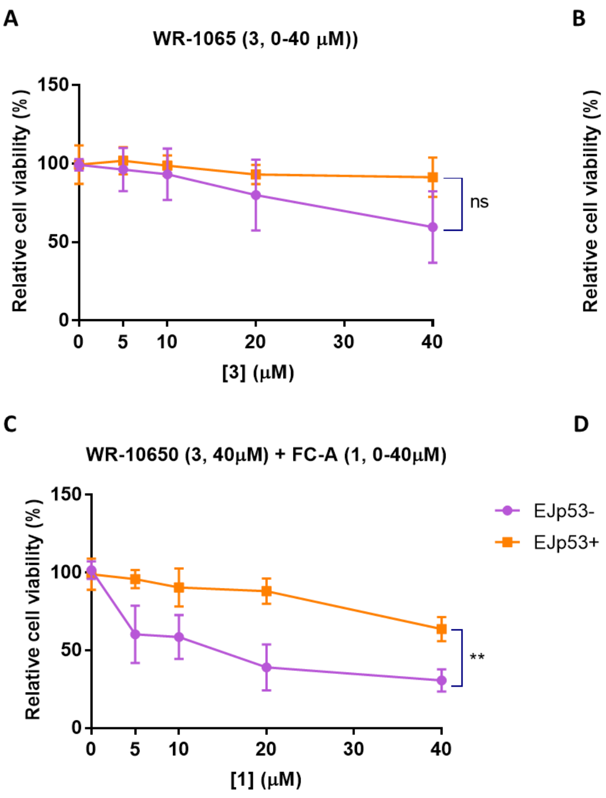

D

IC_{50} (μM)	1 (0-40μM)	3 (0-40μM)	3 (40μM) + 1 (0-40μM)
Ejp53 +	212.4 ± 331	276.7 ± 647	60.02 ± 11.86
Ejp53 -	52.28 ± 10.54	51.54 ± 15.57	11.84 ± 2.61

S10. Treatment of EJp53 cells with 1 and 3 alone and the combination of the two. A-B: EJp53 cells were treated with **1** (0-40 μM) or **3** (0-40 μM). **C:** EJp53 cells were treated with **3** (40 μM) and varying concentrations of **1** (0-40 μM). Metabolic activity was quantified after 72 hours. A T-test statistical analysis was performed. **D:** IC_{50} values for each binding curve. A sigmoidal dose response curve (unequal variance) was used for IC_{50} analysis. All data were normalised to a DMSO treated control. Experiments were performed in technical triplicate and are representative of at least two independent repeats. Error shown is the standard deviation from the mean. ($p < 0.12$ (ns), $p < 0.033$ (*), $p < 0.002$ (**), $p < 0.001$ (***)).

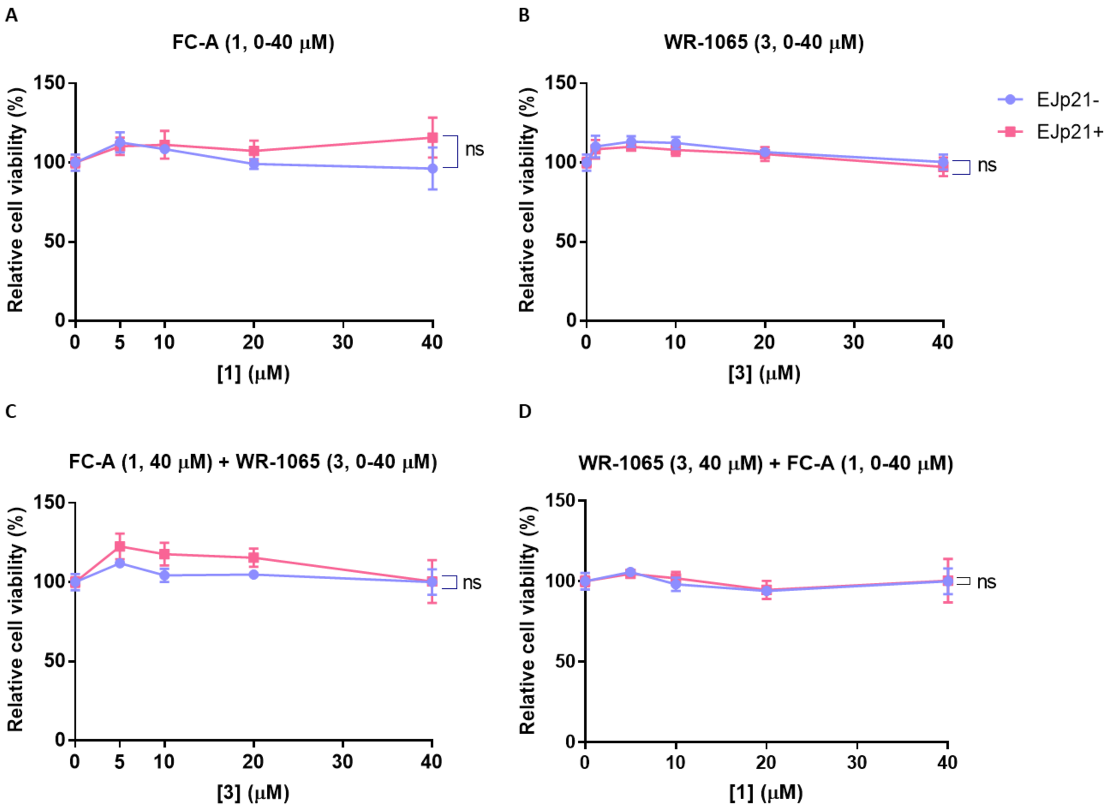

S11. Treatment of EJp21 cells with 1, 3 and combination of the two. A-B: EJp21 cells were treated with **1** (0-40 µM) or **3** (0-40 µM). **C-D:** EJp21 cells were treated with **1** (40 µM) and varying concentration of **3** (0-40 µM), or **3** (40 µM) and **1** (0-40 µM). Metabolic activity was quantified after 72 hours. A T-test statistical analysis was performed. All data were normalised to a DMSO treated control. Experiments were performed in technical triplicate and are representative of at least two independent repeats. Error shown is the standard deviation from the mean. ($p < 0.12$ (ns), $p < 0.033$ (*), $p < 0.002$ (**), $p < 0.001$ (***)).

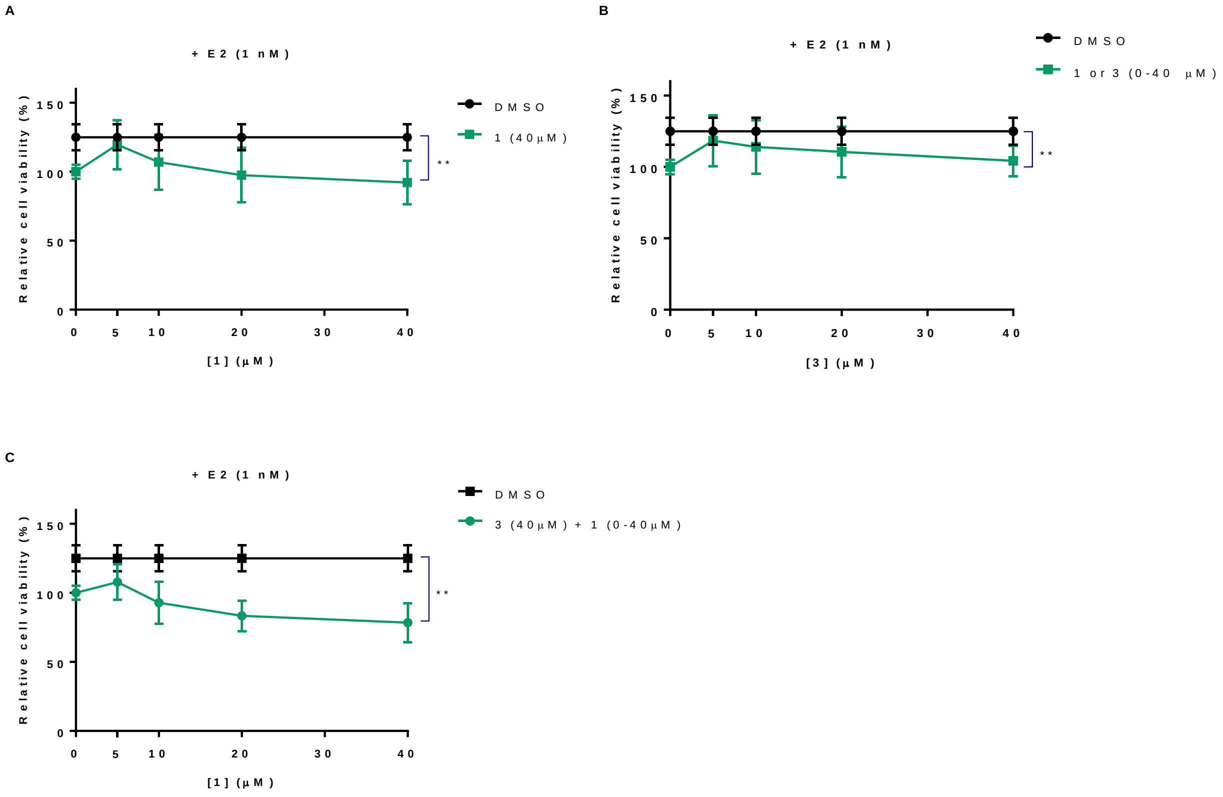

S12. Treatment of E2 induced-MCF-7 cells with 1, 3 and combination of the two. A-B: MCF-7 cells were pre-treated with **1** or **3** alone (0-40 μM) 1h before the addition of E2 (1nM). **C:** MCF-7 cells were pre-treated with **3** (40 μM) and varying concentration of **1** (0-40 μM), 1h before the addition of E2 (1nM). Metabolic activity was quantified after 72 hours. A T-test statistical analysis was performed. All data were normalised to a DMSO treated control. Experiments were performed in technical triplicate and are representative of at least two independent repeats. Error shown is the standard deviation from the mean. ($p < 0.12$ (ns), $p < 0.033$ (*), $p < 0.002$ (**), $p < 0.001$ (***)).

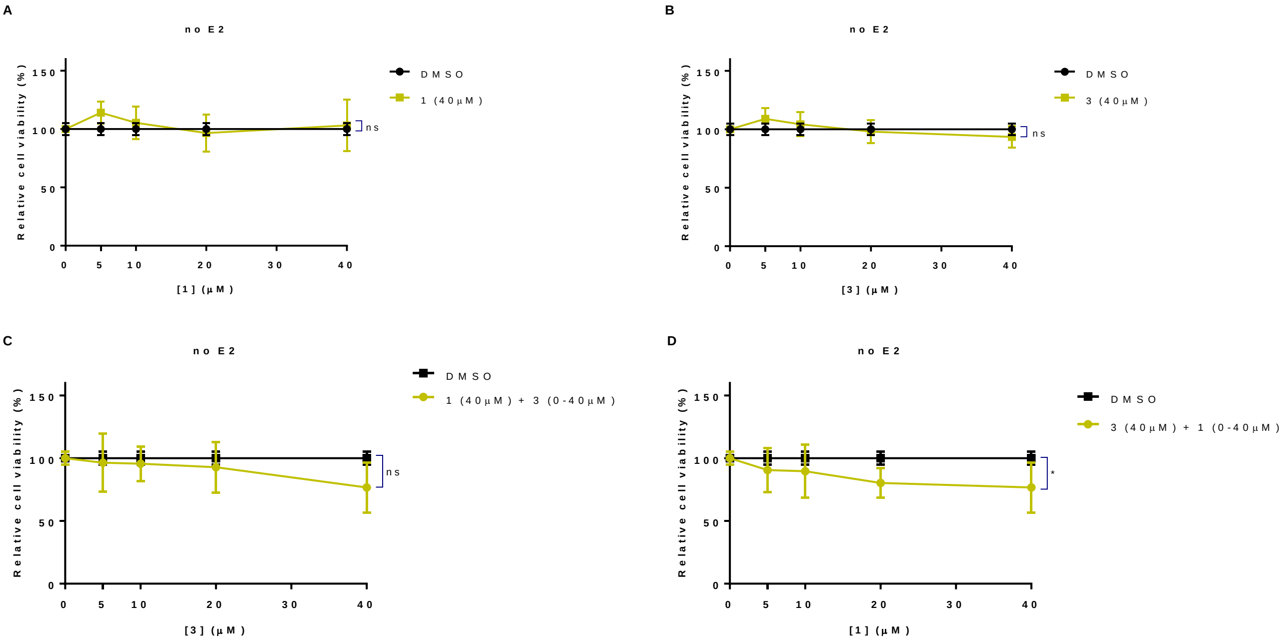

S13. Treatment of MCF-7 cells with 1, 3 and combination of the two. A-B: MCF-7 cells were treated with **1** or **3** alone (0-40 µM). **C-D:** MCF-7 cells were treated with **1** (40 µM) and varying concentration of **3** (0-40 µM) or with **3** (40 µM) and **1** (0-40 µM). Metabolic activity was quantified after 72 hours. A T-test statistical analysis was performed. All data were normalised to a DMSO treated control. Experiments were performed in technical triplicate and are representative of at least two independent repeats. Error shown is the standard deviation from the mean. ($p < 0.12$ (ns), $p < 0.033$ (*), $p < 0.002$ (**), $p < 0.001$ (***)).

Peptide: p53 (pT387)

Sequence: AcHN-^{379}RHKKLMFK(pT)EGPDSD^{393}-COOH

Molecular Weight: 1911.05 g/mol

Purity: 96.65%

ESI-MS m/z: $[M-H]^+$ 1911.89 (see spectra below)

HPLC: Retention Time = 18.48 minutes (linear gradient 5-95 % MeCN in H_20 (0.1 % TFA as an additive) over 30 minutes (see spectra below).

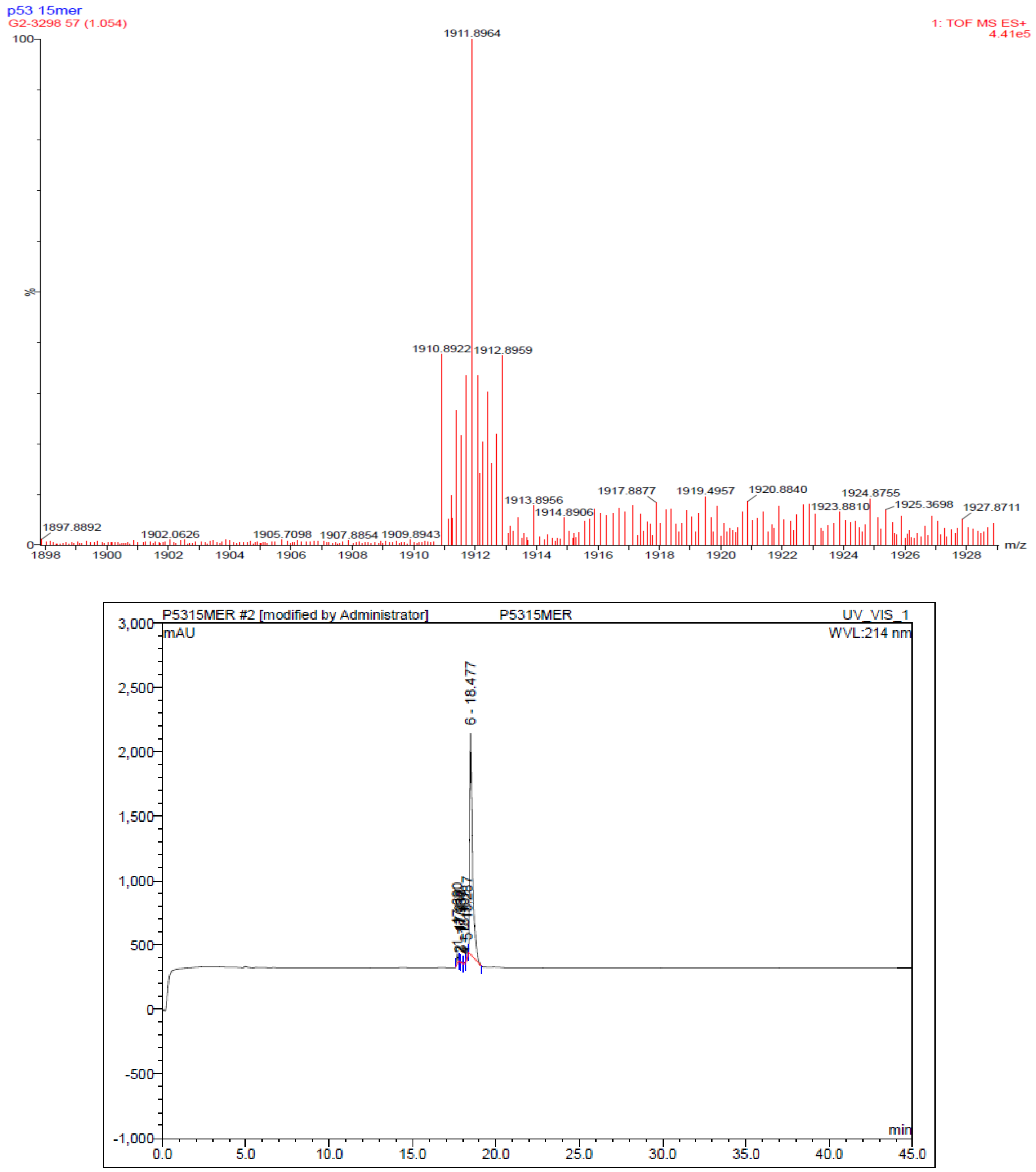

Figure S14A: ESI-MS and HPLC spectra of p53 (pT387) phospo-peptide

Peptide: TAMRA-ERα-8mer (pT584)

Sequence: TAMRA-βA-[577]AEGFPA(pT)V[585]-COOH

Molecular Weight: 1354.40 g/mol

Purity: 96.40%

ESI-MS m/z: $[M-H]^+$ 1355.56 (see spectra below)

HPLC: Retention Time = 23.59 minutes (linear gradient 5-95 % MeCN in H_20 (0.1 % TFA as an additive) over 30 minutes (see spectra below)

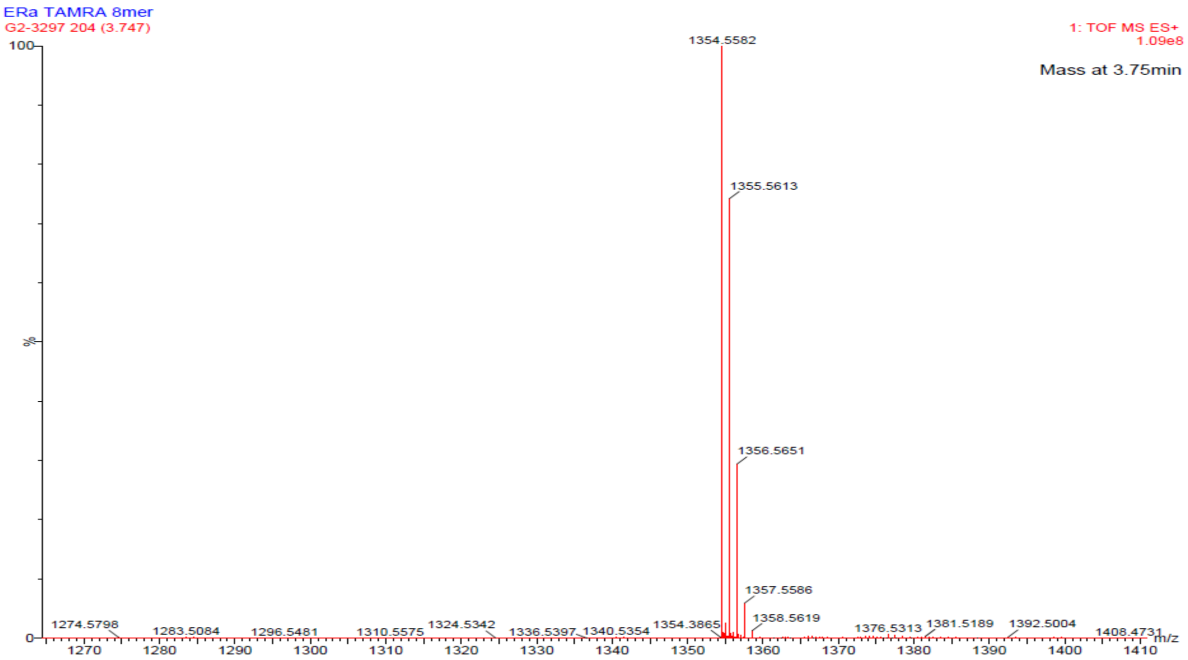

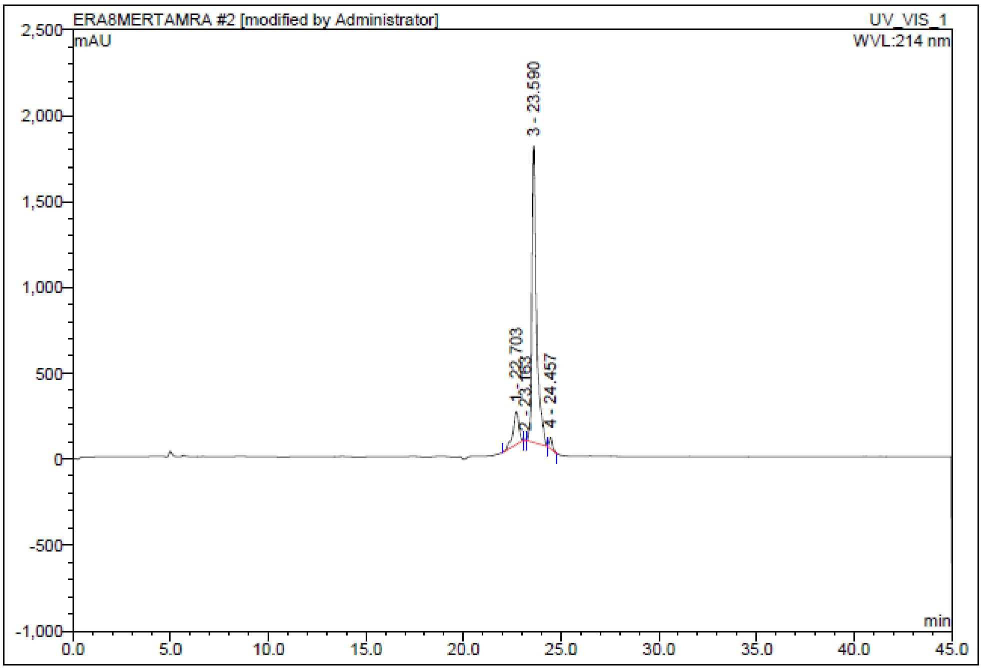

Figure S14B: ESI-MS and HPLC spectra of TAMRA-ERα-8mer (pT584) phospo-peptide.

Peptide: ERα-15mer (pT584)
Sequence: Ac-^{570}KYYITGEAEGFPA(pT)V^{585}-COOH
Molecular Weight: 1767.83 g/mol
Purity: 96.37%
ESI-MS m/z: $[M-H]^+$ 1768.80 (see spectra below)
HPLC: Retention Time = 21.12 minutes (linear gradient 5-95 % MeCN in H_2O (0.1 % TFA as an additive) over 30 minutes (see spectra below).

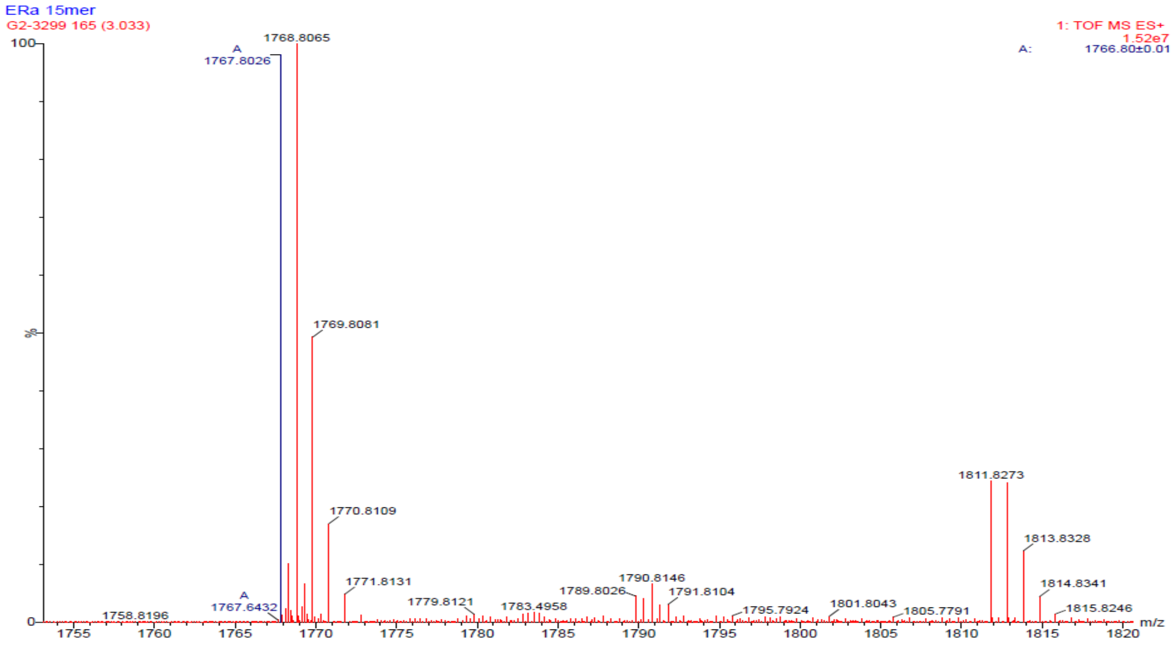

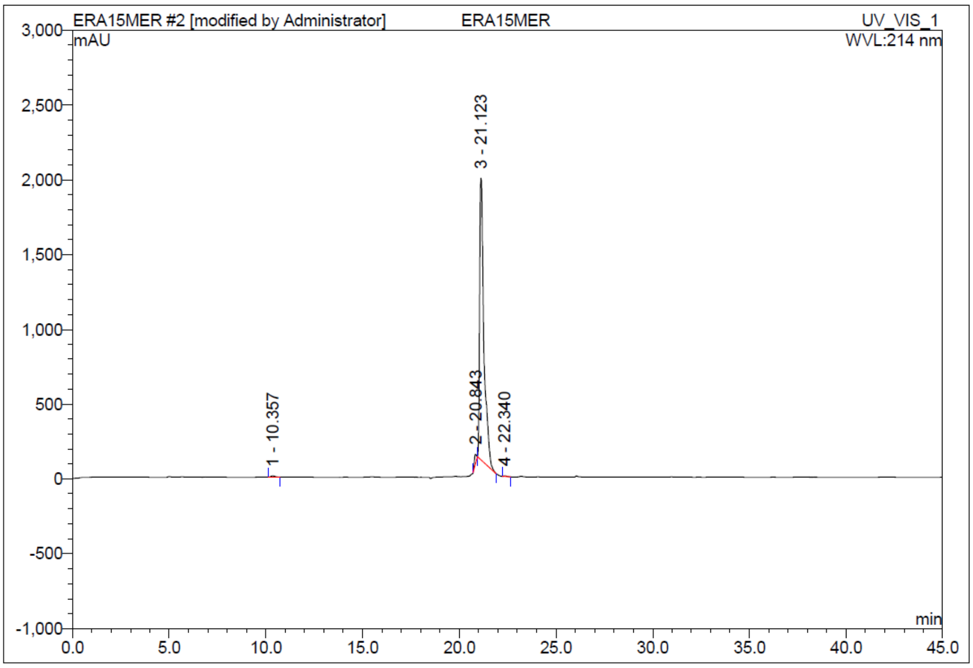

Figure S14C: ESI-MS and HPLC spectra of ERα-15mer (pT584) phospo-peptide.

Peptide: ERα-8mer (pT584)
Sequence: Ac-^{577}AEGFPA(pT)V^{585}-COOH
Molecular Weight: 912.88 g/mol
Purity: 97.27%
ESI-MS m/z: $[M-H]^+$ 913.37 (see spectra below)
HPLC: Retention Time = 20.03 minutes (linear gradient 5-95 % MeCN in $H_2$0 (0.1 % TFA as an additive) over 30 minutes (see spectra below).

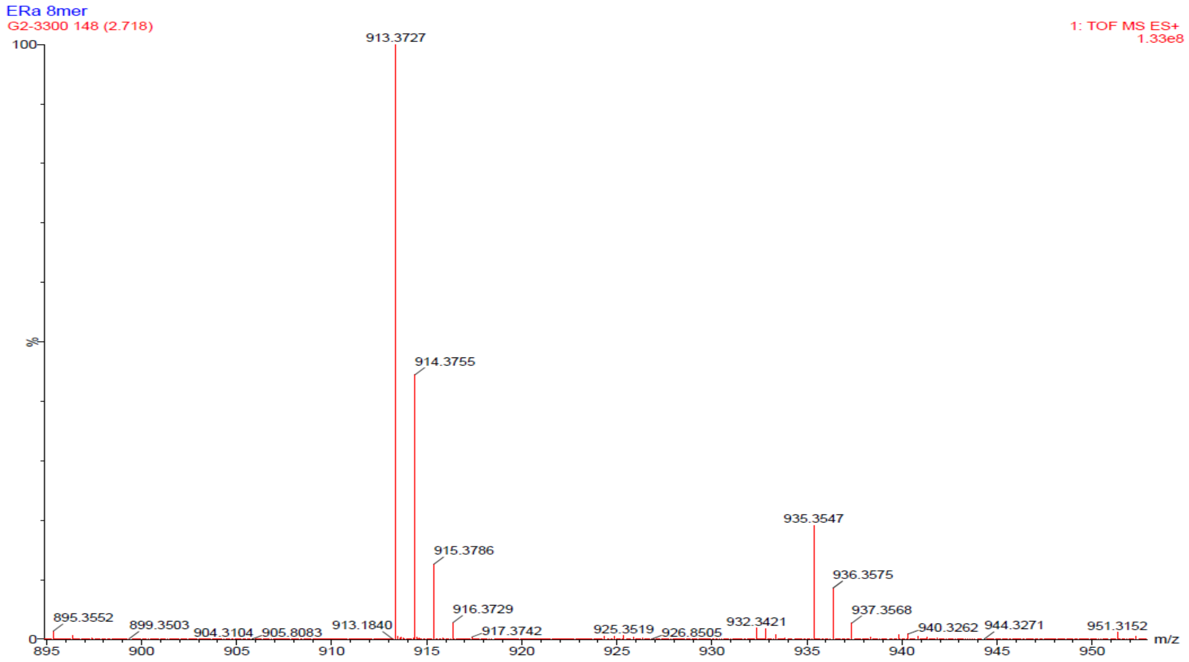

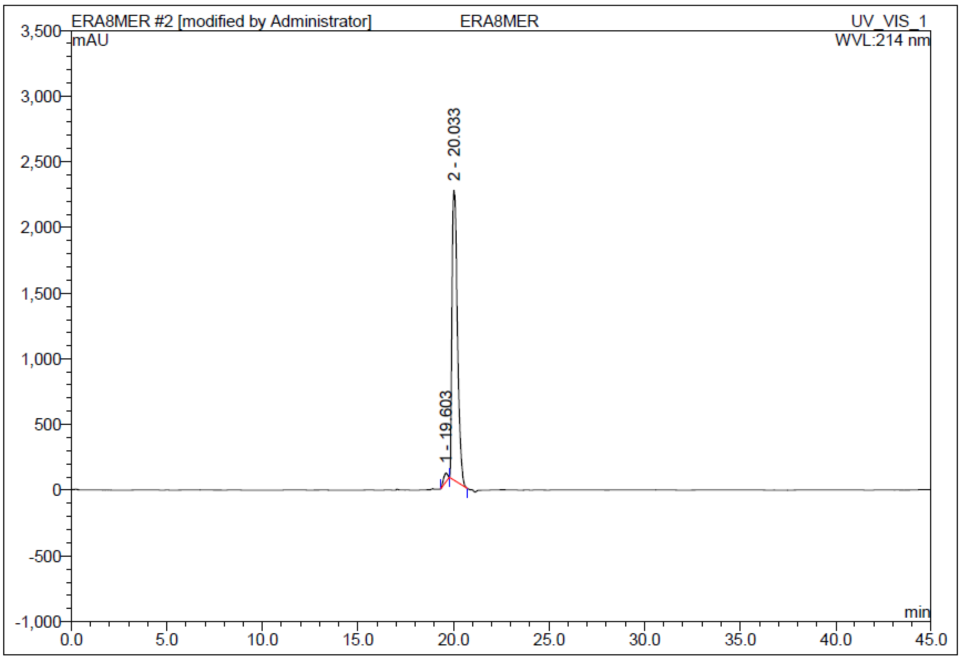

Figure S14D: ESI-MS and HPLC of ERα-8mer (pT584).

Experimental Procedures

Protein Expression

Fluorescence polarisation and isothermal titration calorimetry: Recombinant full length 14-3-3σ/ζ with a TEV protease cleavable N-terminal His-tag was expressed in BL21 (DE3) competent cells with a pPROEX Htb plasmid and purified by Ni^{2+} affinity chromatography (see Figure **S15A)**. The proteins were dialyzed against fluorescence polarisation (FP) or isothermal titration calorimetry (ITC) buffer (25 mM HEPES pH 7.5, 100 mM NaCl, 10 mM $MgCl_2$) before use and concentrated using a centrifugal filter unit (Merck Millipore).

Cys38Ala 14-3-3σ: A plasmid containing a Cys38Ala mutation was generated by quick change mutagenesis using a QuikChange II Site-Directed Mutagenesis Kit (Agilent) using synthetic primers (Eurofins) and according to the manufacturer instructions. The purified plasmid containing the desired mutation was transformed into XL1-Blue supercompetent cells and the DNA obtained was purified using a QIAGEN mini prep kit. The sequence was confirmed by Sanger sequencing performed by SourceBioscience. The 14-3-3σ [Cys38Ala] protein was expressed and purified as described above.

Protein crystallography: A truncated 14-3-3σ construct (14-3-3σ ΔC, aa 1-236) with a TEV protease cleavable N-terminal His-tag was expressed as above. This was purified by Ni^{2+}-affinity chromatography. The His-tag was cleaved using TEV protease and removed by a second iteration of Ni^{2+}-affinity chromatography before dialysis against buffer containing 25 mM HEPES, 100 mM NaCl, 10 mM $MgCl_2$, pH 8.0. The protein was then further purified by size-exclusion chromatography using a Superdex 75 column, eluting with the same buffer (see Figure **S15B)**. Protein-containing fractions were concentrated using a centrifugal filter unit (Merck Millipore).

Individual proteins were analysed by SDS-PAGE after purification and concentration (see Figure **S15A**).

A

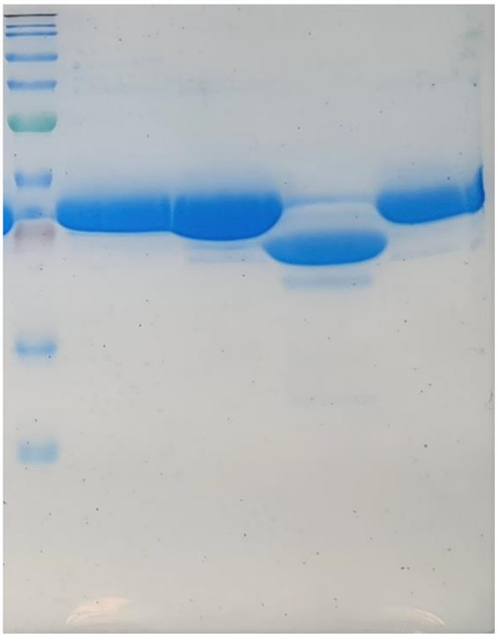

B

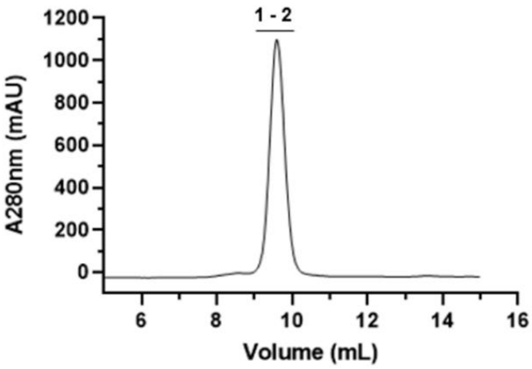

Figure S15: A: SDS-PAGE gel of the individual proteins used in this work: from left to right: ladder (colour prestained protein standard (11 to 245 kDa), 14-3-3ζ, 14-3-3σ, 14-3-3σ ΔC, 14-3-3 C38A mutant. **B:** Size-exclusion chromatography trace of 14-3-3σ ΔC.

Phospho-Peptide Information

Peptide sequences and sources are summarised below in Table S3. HPLC and ESI-MS spectra of peptides used in this work are listed in Figures S8A-D.

Table S3. Phospho-peptide sequences and sources.

Peptide	Sequence	Source
p53 (pT387)	AcHN-^{379}RHKKLMFK(pT)EGPDSD^{393}-COOH	China Peptides
TAMRA-p53 (pT387)	TAMRA-LC-^{361}SRAHSSHLKSKKGQSTSRHKKLMFK(pT)EGPDSD^{393}-COOH	Anaspec
TAMRA-ERα (pT584)	TAMRA-βA-^{577}AEGFPA(pT)V^{585}-COOH	China Peptides
ERα-15mer (pT584)	Ac-^{570}KYYITGEAEGFPA(pT)V^{585}-COOH	China Peptides
ERα-8mer (pT584)	Ac-^{577}AEGFPA(pT)V^{585}-COOH	China Peptides

Peptide: TAMRA-p53
Sequence: TAMRA-Ahc-^{362}SRAHSSHLKSKKGQSTSRHKKLMFK(pT)EGPDSD^{393}-COOH
Molecular Weight: 4200.06 g/mol
Purity: >95%
ESI-MS m/z and **HPLC:** Analytical data for this peptide batch has been previously reported.[1]

Mass Spectrometry

The 14-3-3 proteins and the ligands (Fig. 2, 10 mM in DMSO) were diluted in ITC buffer to give a final protein concentration of 0.1 mM and a final ligand concentration of 0.1 mM (1% v/v DMSO). Samples were incubated at room temperature overnight. 8.0 µL of each sample was diluted with 192 µL H_2O containing 0.1% v/v formic acid to give a final protein concentration of 0.1 mg/mL. Mass spectra were recorded on a Waters Acquity XEVO Q ToF instrument, processed using MassLynx and deconvoluted using the MaxEnt function.

Fluorescence Polarisation Assays

Fluorescence Polarisation (FP) measurements were performed in Corning black, round- bottom, low-binding 384-well plates using FP buffer containing 10 mM HEPES pH 7.5, 150 mM NaCl, 0.1% (v/v) Tween20, 0.1% (m/v) BSA. Plates were incubated at room temperature for 30 min and shaken for 10 seconds before measurement using a Hidex Sense Microplate Reader with an excitation wavelength of λex: 535 /20 nm; at an emission wavelength of λem: 590 /20 nm; mirror: dichroic 560; flashes: 20; PMT voltage 750; Z-position: calculated from well. FP data were analysed in GraphPad Prism 7 and sigmoidal curves were fitted using the Levenburg-Marquardt iteration algorithm.

Isothermal Titration Calorimetry

Isothermal Titration Calorimetry (ITC) experiments were conducted with a Malvern MicroCal ITC_{200} instrument in buffer containing: 25 mM HEPES pH 7.5, 100 mM NaCl, 10 mM $MgCl_2$. All measurements were carried out under the following conditions: 25 ^{o}C, 750 rpm stirring speed, reference power 5 µCal/sec, initial spacing of 240 seconds, initial delay of 60 seconds, 2 x 18 injections of 2 µL with 180 seconds of spacing. p53/ERα peptides (1.0/0.5 mM) were titrated to 14-3-3 proteins (0.1 mM) in the cell. Both cell and titrant contained 2% DMSO (v/v) throughout. Ligands were added to a final concentration of 0.1 or 1.0 mM. The CONCAT32 software was used for merging the two titration series and Origin software was used for data analysis (single site binding model) to obtain thermodynamic parameters.

Protein crystallisation

For crystallisation of the 14-3-3σ/ERα 8-mer peptide complex, protein and peptide were mixed in 1:1.5 molar ratio and incubated for 30 min in 25 mM HEPES pH 7.5, 100 mM NaCl, 10 mM $MgCl_2$ to reach a final concentration of 10 mg/mL. Vapour diffusion crystallisation 200 nL sitting drops were set up using a Mosquito crystallisation robot (SPT Labtech), mixing the 14-3-3σ/Erα 8-mer peptide complex in mother liquor in the following volume ratios: 1:1 (drop 1) and 1:2 (drop 2), with the 96-conditions MORPHEUS crystallisation screen (Molecular Dimensions).[2] Crystals grew in drop 1 and 2 within a week at the following conditions: 1 M HEPES pH 7.1-7.3, 0.19 M $CaCl_2$, 29% PEG 400, 5% glycerol, at 4 °C.

The complexes formed by 14-3-3σ/ERα 8-mer peptide and the compounds WR-1065 and FC-A were obtained via a soaking procedure within 3-5 days at 4 °C. A DMSO stock solution of FC-A and WR-1065 were diluted in mother liquor first and then transferred to the drop with the crystals in order to obtain the desired concentrations. The first 14-3-3σ/ERα-8mer crystal was soaked with WR-1065 40 mM for two hours; the second one with an equimolar mixture of WR-1065 and FC-A (both 10 mM, overnight). The crystals were flash cooled in liquid nitrogen and exposed to X-rays.

X-ray data diffraction collection and processing

X-ray diffraction data were collected at beamline I24 (λ= 0.99990 Å and beam size of 50x50 μm^2) at the Diamond Light Source in Harwell, England, UK. Other data collection parameters are listed in Table 1. X-ray diffraction data were processed with the autoPROC suite[3] of programs version 1.0.5 (indexing and integration in XDS[4] and scaling in aimless[5] 0.7.4 followed by anisotropic scaling in STARANISO[6] 2.3.46).

Structure determination and refinement

Initial phases for the structure factor amplitudes of the 14-3-3σ/ERα-8mer crystal soaked with WR-1065 were computed by rigid-body refinement starting from the PDB ID 4JC3 model (the published structure of the 14-3-3σ/ERα-8mer binary complex); while initial phases for the structure factor amplitudes of the 14-3-3σ/ERα-8mer crystal soaked with WR-1065/FC-A were computed by rigid-body refinement starting from the PDB ID 4JDD model (the published structure of the 14-3-3σ/ERα-8mer/FC-A ternary complex) using the program CCP4-Molrep version 11.7.02.[7] Refinement statistics data are listed in Table 2.

Cell lines and treatment

MCF-7, EJp53[8] and EJp21[9] adherent cells were maintained in Dulbecco's modified Eagle's medium supplemented with 10% fetal bovine serum, penicillin-streptomycin (50 units/ml). For EJ cells the following antibiotics were added to the culture media: hygromycin (100 µg/ml), geneticin (750 µg/ml) and tetracycline (TET) (1 µg/ml) every 2-3 days. Cells were kept in a 37°C incubator at 5% of CO_2. To induce p53 and p21 expression, EJ cells were washed three times with phosphate-buffered saline (PBS), spun down in the centrifuge (200 g for 3 min) and seeded in medium in absence of TET.

Detection of senescence-associated β-galactosidase (SA-β-gal)

SA-β-gal was performed according to Judith Campisi's Lab protocol.[10] Senescent and proliferating cells were washed twice with PBS before and after being fixed with 10% neutral buffered formalin at room temperature. Plates were incubated for 48-72h at 37°C without CO_2 with a staining solution containing the following reagents: 1 mg/ml 5-bromo-4-chloro-3-indolyl-β-D-galactopyranoside, 5 mM potassium ferrocyanide, 5 mM potassium ferricyanide, 150 mM NaCl, 2 mM MgCl2, and 0.1 M phosphate buffer, pH 6.0. Cells were observed under the microscope looking for stained blue cells which indicate a positive SA-b-gal assay.

CellTiter-Glo Viability Assay

Cell lines were seeded at $4x10^4$ cells per 100µl/well in a 96-well plate in triplicates and treated with FC-A or WR-1065 and with a combination of the two compounds at concentrations varying between 0-40 µM. Plates were incubated for 72h and cell viability was measured using CellTiter-Glo (Promega G7572). 5-10% v/v of the reagent was added to each well and incubated for 5 minutes in the dark at room temperature before the luminescent signal was measured using a HidexSense plate reader.

Statistical Analysis

Data were normalised to control samples (treated with an equal volume of DMSO). Cell line experiments were performed in technical triplicates with a minimum of two biological replicates and expressed as ± standard error of the mean.

GraphPad Prism was used for the statistical analysis. Paired t-test (non-parametric) was performed to assess differences between EJ senescent cells and EJ proliferating cells (control). The differences between treatment groups were assessed by using One-way ANOVA with Dunnett's post hoc test (*$p < 0.02$, **$p < 0.005$, ***$p < 0.001$). The half-maximal response (IC_{50}) to drugs were calculated by using a sigmoidal dose response curve (unequal variance) based on CTG data with upper and lower constraints set to 100 and 0%.

References

1. Andrei, S.A., de Vink, P., Sijbesma, E., Han, L., Brunsveld, L., Kato, N., Ottmann, C., Higuchi, Y., *Angew. Chem. Int. Ed.* 2018, **57**, 13470-13474.

2. Gorrec F: The MORPHEUS protein crystallization screen. *J Appl Crystallogr.* 2009; **42**(Pt 6), 1035–1042.

3. Vonrhein, C., Flensburg, C., Keller, P., Sharff, A., Smart, O., Paciorek, W., Womack, T. & Bricogne, G. (2011). Data processing and analysis with the autoPROC toolbox. *Acta Cryst.* **D67**, 293-302.

4. Kabsch W. XDS. 2010 Feb. *Acta Cryst.* **D66**(Pt 2), 125-32.

5. Evans, P.R. & Murshudov, G.N. (2013). How good are my data and what is theresolution?, *Acta Cryst.* **D69**, 1204-1214.

6. Tickle, I.J., Flensburg, C., Keller, P., Paciorek, W., Sharff, A., Vonrhein, C., Bricogne, G. (2018). STARANISO. Cambridge, United Kingdom: Global Phasing Ltd.

7. Winn, M.D., Ballard, C.C., Cowtan, K.D. Dodson, E.J., Emsley, P., Evans, P.R.,Keegan, R.M., Krissinel, E.B., Leslie, A.G.W., McCoy, A., McNicholas, S.J.,Murshudov, G.N., Pannu, N.S., Potterton, E.A., Powell, H.R., Read, R.J., Vagin,A. & Wilson, K.S. (2011). Overview of the CCP4 Suite and Current Developments. *ActaCryst*, **D67**, 235-242.

8. Sugrue, M. M., Shin, D.Y., Lee. S.W., Aaronson, S. A., *Proc. Natl. Acad. Sci.*, 1997, **94**, 9648–9653.

9. Fang, L., Igarashi, M., Leung, J., Sugrue, M.M., Lee, S.W., Aaronson, S.A., *Oncogene,* 1999, **18**, 2789-2797.

10. Dimri, G. P., Lee, X., Basile, G., Acosta, M., Scott, G., Roskelley, C., Medrano, E. E., Linskens, M., Rubelj, I., Pereira-Smith, O., Peacocke, M., Campisi., J., *Proc. Natl. Acad. Sci.* 1995, **92**, 9363-9367.
